# Development of a Microwave-assisted Chemoselective Synthesis of Oxime-linked Sugar Linkers and Trivalent Glycoclusters

**DOI:** 10.3390/ph12010039

**Published:** 2019-03-14

**Authors:** Katherine McReynolds, Dustin Dimas, Grace Floyd, Kara Zeman

**Affiliations:** Department of Chemistry, California State University, Sacramento, 6000 J Street, Sacramento, CA 95819-6057, USA; Dustin.Dimas-1@ou.edu (D.D.); gracepfloyd@gmail.com (G.F.); karazeman@csus.edu (K.Z.)

**Keywords:** Microwave reactions, chemoselective, oxime, aminooxy, glycoclusters, multivalent

## Abstract

A rapid, high-yielding microwave-mediated synthetic procedure was developed and optimized using a model system of monovalent sugar linkers, with the ultimate goal of using this method for the synthesis of multivalent glycoclusters. The reaction occurs between the aldehyde/ketone on the sugars and an aminooxy moiety on the linker/trivalent core molecules used in this study, yielding acid-stable oxime linkages in the products and was carried out using equimolar quantities of reactants under mild aqueous conditions. Because the reaction is chemoselective, sugars can be incorporated without the use of protecting groups and the reactions can be completed in as little as 30 min in the microwave. As an added advantage, in the synthesis of the trivalent glycoclusters, the fully substituted trivalent molecules were the major products produced in excellent yields. These results illustrate the potential of this rapid oxime-forming microwave-mediated reaction in the synthesis of larger, more complex glycoconjugates and glycoclusters for use in a wide variety of biomedical applications.

## 1. Introduction

Multivalent glycoconjugates such as glycoclusters, glycodendrimers, glyconanoparticles and glycan microarrays have found a wide range of applications in biochemistry and medicine from studying protein-carbohydrate interactions to developing potential therapeutic agents [1,2,3,4]. It has long been recognized in nature that proteins and their binding partners are often displayed in a multivalent fashion. It is also well known for carbohydrate-containing molecules that their binding interactions with their biological partners are much stronger when present in multiple copies rather than in a 1:1 ratio. This is known as the multivalent or cluster glycoside effect [2,3,5,6].

Given the complexity and size of multivalent glycoconjugates, as well as the requirement for multiple simultaneous reactions to append the sugars, it is critical that a synthetic strategy be developed to create these molecules in an efficient and fully functionalized manner. Commonly, carbohydrate chemistry requires multiple protection/deprotection steps due to the similar reactivity of the hydroxyl groups present. This can lead to time-consuming reaction pathways with low yielding final products. Some studies have sought to simplify the attachment of the carbohydrates to the multivalent scaffold. We previously showed that we could use amide coupling between the carboxylic acid-containing unprotected sugar, sialic acid and a poly(amidoamine) (PAMAM) dendrimer core to create a fully substituted 2nd generation 16-mer glycodendrimer that showed µM activity against HIV-1 [7]. However, this strategy is only applicable to sugars with either an existing amine or carboxylic acid group or would require the introduction of these groups, leading to further steps. Another approach uses click chemistry, however, this still requires functionalization of the carbohydrate moiety with either an alkyne or an azide group, requiring a multi-step synthetic process involving protecting group chemistry to install those groups [2,8,9,10].

**Figure 1:**
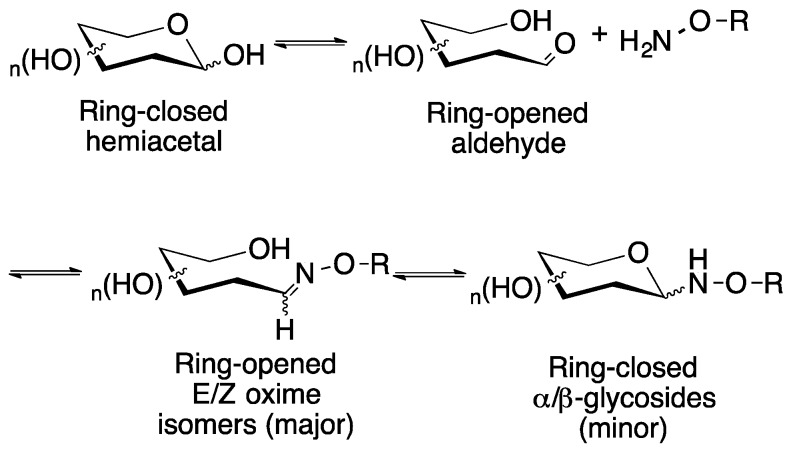
Reaction of a typical reducing aldose sugar with an aminooxy-containing compound in a protic solvent such as water results in the initial formation of the ring-opened E/Z oximes, which over time in aqueous solution, will equilibrate with the ring closed glycosides.

As an alternative strategy to either amide or click chemistry, we sought a streamlined synthetic process that would allow us to use unprotected carbohydrates in the coupling step to a multivalent scaffold molecule to create the desired fully substituted glycoclusters. This involved a two-part simplification strategy. First, a chemoselective oxime-forming reaction was selected [11,12]. Oxime linkages are known to be both acid- and glycosidase-stable, making them more robust than glycosidic linkages for biological applications [13,14,15]. The oximation reaction occurs under mild, aqueous conditions between an aminooxy-containing molecule, here a monovalent linker or a multivalent core [16] and an unprotected reducing aldose (hemiacetal) or ketose (hemiketal) sugar [17,18]. Oxime linkages in sugars can exist in an equilibrium mixture containing two forms in protic solutions, the ring opened oxime, with E/Z isomers (major products) and the ring-closed glycoside, comprised of the α- and β-anomers (minor products, Figure 1) [11,19]. The second simplification to the synthetic process involved the use of a microwave-mediated reaction for the formation of the oxime linkage. This was done for multiple reasons. First, to shorten the reaction times from several hours down to minutes in duration [20]. Next, to synthesize the desired glycoconjugates in good yields and for the multivalent glycoclusters, to ensure that simultaneous reactions of each sugar with the complementary aminooxy group on the linker/core molecule could be achieved. Finally, to simplify the process and ensure reproducibility of the reaction through the use of programmed methods. These characteristics of microwave-mediated reactions make them particularly attractive for the synthesis of carbohydrate-containing molecules, which can be sensitive to long and harsh reaction conditions [21]. Additionally, there are only a few reports of microwave-assisted reactions for the purpose of synthesizing multivalent glycoconjugates [22,23,24,25,26,27,28,29,30]. Most of these reports focus on the use of microwave irradiation to mediate click chemistry reactions. There is only one reported use of alternate microwave-mediated reactions to form multivalent oxime-linked glycopeptoids [30]. In this paper, Carrasco and coworkers reported the microscale microwave-assisted synthesis of a glycopeptoid using a 50 to 100-fold excess of sugar.

Other traditional, non-microwave-mediated studies similarly show the use of multiple equivalents of sugar relative to oxime forming partners. For simple monovalent systems, 2–3 equivalents of aldose sugar were reacted with aminooxy-containing linkers/peptides, both in the presence/absence of aniline for 1–48 h at 25 °C or 60 °C [31]. Here, Jensen and coworkers reported yields for the GlcNAc oxime linker as 7 and 72% for a 1-h reaction conducted at 25 or 60 °C., respectively, for the non-aniline catalyzed reaction, illustrating that elevated temperatures alone improved reaction rates. For the same GlcNAc reaction conducted with 0.1 M aniline included, the yields were 20 and 80% for a 1 or 6 h reaction time at 25 °C, respectively [31]. For the ketose sialic acid sugars, Szabo and coworkers reported the synthesis of sialic acid and tetrasialic acid conjugated to one side of a di-aminooxy linker [32]. The reactions were carried out at 37 °C for 22 h using 19 equivalents of the linker to the sugar to avoid crosslinking. These reactions resulted in yields of 33 and 50%, respectively, for sialic acid and tetrasialic acid-linked monovalent conjugates. Finally, for an example of a traditional multivalent oxime-forming reaction, Renaudet et al. reported yields of 66, 65 and 74% for the syntheses of tetravalent oxime-linked glycopeptoids using the anomeric aminooxy-sugars α-Fuc, β-Fuc and β-Gal and a tetra-aldehyde-bearing cyclic peptide. The reactions were conducted at 37 °C for 2 h and utilized 2 equivalents of sugar per reactive site [33].

Here we report the development of an efficient microwave-mediated method used to synthesize both mono- and multivalent oxime-linked linkers and glycoclusters, respectively. Our method uses *equimolar* ratios of sugar:aminooxy-linker/core and can be completed in as little as 30 min of total reaction time, such that many reactions can be completed in the space of a day and precious/rare glycans used sparingly. This microwave procedure is also simple to set up and operate, making it possible for the rapid production of a wide variety of glycoconjugates by junior researchers/technicians in the lab. Reaction condition uniformity can also be maintained through the use of a programmed method, thereby increasing method consistency and minimizing trial-to-trial variation. Through our microwave-mediated procedure, we have been able to demonstrate both the preparative production of sugar-linkers in a single step, such that they can be isolated and utilized in further synthetic transformations or the facile synthesis of novel glycoclusters in excellent yields that can then be readily incorporated into biological studies. Finally, we have also illustrated that we can create large quantities of the sugar-linker conjugates using our microwave-mediated conditions in good yields without the addition of the common catalyst, aniline [31,34]. Removal of the aniline catalyst and using the microwave to shorten the reaction time both lend themselves to making the reaction greener overall.

## 2. Results

**Scheme 1:**
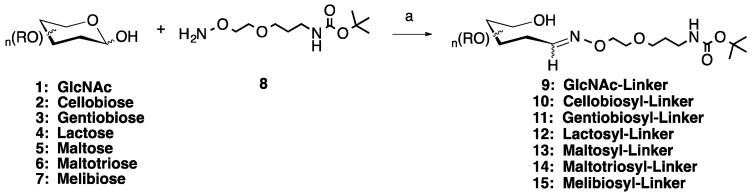
Microwave synthesis of sugar linker conjugates **9**–**15**. (a) 0.1 M NH_4_OAc, pH 4.5, 25% of 400 W, 50 °C, 30 min. Optional: 0.1.

In this paper we outline the successful combination of chemoselectivity with a microwave-mediated reaction for purposes of synthesizing a series of oxime-linked monovalent sugar-linker molecules and three trivalent glycoclusters. We first evaluated seven common aldose mono-, di- and tri-saccharides (*N*-acetyl glucosamine, cellobiose, gentiobiose, lactose, maltose, maltotriose and melibiose, **1–7**, Scheme 1) for the preparation of the monovalent sugar linkers. This chemistry was undertaken to determine the optimum microwave reaction conditions necessary for the reaction in equimolar quantities of sugar to aminooxy-linker. The study had a goal of minimizing the use of excess reactants, particularly if expensive/difficult to create sugars were to be used, which would also serve to simplify the purification process. Traditionally, aniline is used as a catalyst in oxime-forming reactions because it yields significant increases to the reaction rate [31,34]. Therefore, in our development of the microwave-mediated method, we evaluated whether or not aniline was required to improve the yields for oxime formation or whether it could be omitted to make the reaction greener and easier to purify, without significantly sacrificing the reaction yield. The resultant sugar-linker molecules are useful intermediates in the development of multivalent glycoconjugates. The trivalent glycoclusters synthesized in this study provide proof of concept for the synthesis of higher order glycoclusters efficiently and excellent isolated yields via a chemoselective microwave synthesis.

**Scheme 2:**
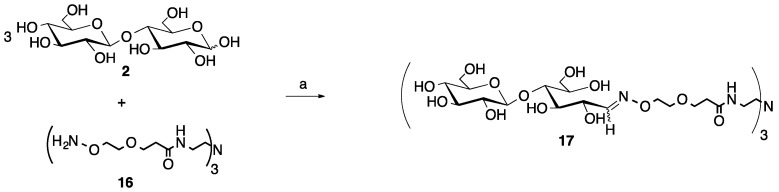
Microwave synthesis of trivalent cellobiose glycocluster (17, 94% yield). (a) 0.1 M NH_4_OAc, pH 4.5, 0.1 M aniline, 25% of 400 W, 30 min.

**Scheme 3:**
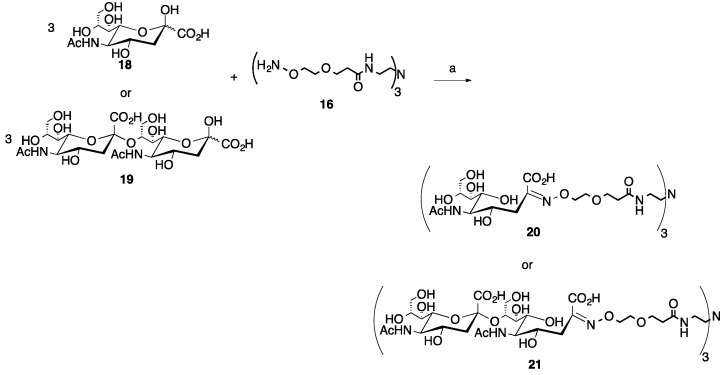
Microwave synthesis of trivalent sialic acid (20, 82%) and disialic acid (Sia(α-2→8)Sia, 21, 88%) glycoclusters. (a) 0.1 M NH_4_OAc, pH 4.5, 0.1 M aniline, 25% of 400 W, 30–90 min.

For the trivalent glycocluster synthesis, we employed both an aldose reducing disaccharide, cellobiose (**2**, Scheme 2) and two ketose sugars, sialic acid (*N*-acetyl neuraminic acid, Neu5Ac, **18**, Scheme 3) and the α-2→8-linked dimer of sialic acid (disialic acid) (**19**, Scheme 3) [35]. These sugars were chosen to illustrate that the microwave reaction worked efficiently for both types of sugars and that the glycosidic bonds present in **2** and **19** would be stable to the microwave heating conditions at a pH of 4.5. Sialic acid-containing glycans are widely found in nature and are important markers in disease states such as cancer, influenza and meningococcal meningitis [36,37]. The produced glycoclusters contain the acid/glycosidase stable oxime linkage, which can help ensure the integrity of the molecules if they are ultimately used in biological applications [13,14,15]. It is also worth noting that longer oligosaccharides may be necessary given that the reducing end sugar will exist as a mixture of the native closed ring conformation and the open ring oxime, which may impact the resultant biological activity.

### 2.1. Medium Scale Microwave-mediated Synthesis of Monovalent Sugar-Linkers

To begin the medium scale (≤0.250 mmol) synthesis of the monovalent sugar linker molecules, a bifunctional, hydrophilic aminooxy-Boc-protected amine linker was used (**8**, Scheme 1) [16]. This was combined 1:1 with any of the seven off-the-shelf aldose mono-, di- and tri-saccharides (1–7) in 0.1 M ammonium acetate, pH 4.5, in the presence or absence of 0.1 M aniline. The reactions were carried out in a CEM MARS 5 laboratory-grade microwave. Many different combinations of power level and time were attempted, with the optimum combination found to be 30 min at 25% of 400 W, with a 50 °C maximum temperature. Upon completion of the reaction, the solution was freeze dried then purified by flash chromatography on silica gel in a 6:4:0.5 mixture of chloroform:methanol:water. Examination of the pooled fractions by ^1^H NMR showed no evidence of degradation of either the products or unreacted starting materials (See Supplementary Materials for details). This initial set of reactions, using the aniline catalyst, gave rise to 73–93% yields of the Boc-protected sugar linker products (**9–15**, Table 1), while the same reactions conducted without the aniline catalyst resulted in yields ranging from 60–68%, a decrease of 9–28%, depending on the sugar used.

For further comparison, two traditional, non-microwave-mediated reactions to yield the Boc-protected cellobiosyl-linker (**10**) were carried out at 50 °C for 30 min in the presence or absence of the aniline catalyst. Cellobiose was chosen for the sugar, as it represents a typical aldose disaccharide. For the aniline-catalyzed reaction, a 65% yield of **10** resulted, while for the non-catalyzed reaction a yield of 56% of **10** was observed (Table 1). Comparing these results against **10** synthesized in the microwave, modest yield improvements were noted in the microwave mediated reactions. The microwave aniline-catalyzed reaction gave rise to an 11% higher yield and the non-aniline catalyzed microwave reaction resulted in a 7% higher yield. Overall the average % yield increase for all 7 aldoses for the microwave versus traditional reactions in the presence of aniline was 16% and 9% for the non-aniline catalyzed reactions. Interestingly, the average magnitude of the difference between aniline versus non-aniline catalyzed reactions was greater (~15%) for the microwave-mediated reactions than for the traditional heated reactions (9%). These results indicate that modest improvements of yield can be gained by using microwave-mediated conditions, using equimolar quantities of sugar and linker. In addition, if desired, it was found that the aniline catalyst could be left out of the reaction mixture to simplify product purification and make the reaction greener, all without unreasonable yield losses.

### 2.2. Large Scale Microwave-mediated Synthesis of Monovalent Sugar-Linkers

To further evaluate reaction scalability, the microwave reaction was then carried out on the same seven aldose sugars at higher quantities (≥0.800 mmol) using identical microwave reaction conditions as described above. All of the reactions included the 0.1 M aniline catalyst to maximize the yields. Here it was found that the isolated yields in the large-scale reactions ranged from 60–79%, a decrease of 1–30% compared to the medium scale aniline-catalyzed reactions, again depending on the sugar incorporated (Table 1). We noted that the decreases in the yields for the medium scale reactions in the absence of aniline were similar to the decreases seen in the larger scale microwave-mediated reactions in the presence of the aniline catalyst. This means that while the microwave can be an excellent tool for shortening the reaction times for these reactions, a balance must be struck between reaction scale and reaction time savings. For our purposes, it made sense to significantly scale up the reactions, given that the sugars used were all commercially available and the linker could be produced efficiently in large quantities as well [16]. These new monovalent sugar-linker molecules are useful intermediates that can be utilized in the synthesis of further glycoconjugates. Once the Boc group is removed, the resultant amine can be used in amide coupling reactions to attach the sugar linker to whatever carboxyl-containing scaffold/surface is desired.

### 2.3. Multivalent Glycocluster Microwave-mediated Synthesis

Based on the results for the model monovalent sugar linkers, we moved into the microwave-mediated synthesis of multivalent glycoclusters. With multivalent scaffolds, in addition to the desired fully functionalized product (here the trisubstituted glycocluster), under-substituted products are possible (un-, mono- and disubstituted glycoclusters). It was hypothesized that by utilizing our best reaction conditions developed for the equimolar system described above (25% of 400 W power, 30 min, 0.1 M aniline), that the production of under-substituted products would be limited. The synthesis of three novel trivalent glycoclusters was undertaken beginning with the optimized conditions and included one aldose disaccharide, cellobiose (**2**, Scheme 2), as well as a ketose monosaccharide sialic acid (**18**, Scheme 3) and a ketose disaccharide, α-2→8-disialic acid (**19**, Scheme 3), with a previously synthesized trivalent aminooxy-terminated hydrophilic core (**16**) [16]. Here, cellobiose was chosen as a representative aldose disaccharide and both sialic acid and α-2→8-disialic acid [35] were chosen to represent more hindered, less reactive ketose substrates to show the utility of this method for these interesting, biologically important sugars.

Beginning with a 3:1 ratio of cellobiose to the trivalent core (**2** and **16**, respectively), the 30-min reaction time was sufficient to produce only the desired trivalent product, **17**, in 94% yield following purification via size exclusion chromatography (SEC, Scheme 2). This reaction was carried out on a 10-fold lower scale than the monovalent, aniline-catalyzed reaction, which is one possible reason why the yields were higher. No under-substituted products (un-, mono- or disubstituted), sugar degradation or unreacted starting materials were observed for this reaction by ^1^H NMR upon purification.

For the ketoses, the sialic acid (**18**) reaction with the trivalent core (**16**, Scheme 3), a 3:1 ratio of sugar to core was utilized. The reaction was carried out as described above for 30 min and after purification by SEC, an 82% yield of the desired trivalent product (**20**) was achieved. However, unlike the reaction to produce **17**, where no under-substituted products were produced, the disubstituted byproduct was isolated from a separate peak from the SEC purification and identified by ^1^H NMR. Carrying this method forward, the disaccharide ketose, disialic acid, **19**, was reacted in a 3:1 sugar to core (**16**) ratio under the same conditions as used for sialic acid. However, it was noted that 30 min was not sufficient to achieve a good yield for the desired trivalent product, **21**. This is likely due to steric issues, so two additional 30-min cycles were carried out under the same conditions for a total of 90 min of microwave reaction time. After purification by SEC, an 88% yield of the desired trivalent product (**21**) was achieved. Similar to the sialic acid reaction, the disubstituted byproduct was isolated from a separate peak after SEC purification and was identified by ^1^H NMR. No other under-substituted products or other byproducts were noted from the pooled column fractions for any of the observed peaks, showing again that the microwave-mediated reaction conditions are mild enough to use for these more expensive sugars.

## 3. Discussion

In conclusion, the development of an efficient microwave-mediated oxime forming reaction between equimolar quantities of unprotected aldose or ketose sugars and either a monovalent or trivalent aminooxy-containing linker or core was undertaken with the aims to create a facile and reproducible method that resulted in decreased reaction times, minimization of the amounts of sugars/catalysts used to reduce costs and make the reactions greener, while still creating the desired glycoconjugates efficiently and in good to excellent yields. We began our studies with the formation of model monovalent oxime-linked sugar linker molecules at two different preparative reaction scales (medium and large). This was done to determine the optimum microwave-mediated conditions necessary to achieve the best yields in the smallest amount of time without requiring an excess of either the sugar or linker molecules. Once this was accomplished, the microwave reaction conditions were then applied to more complex multivalent systems, such that biologically relevant glycoclusters could be prepared in their desired fully substituted forms in a matter of minutes in a single chemoselective step.

To begin with the monovalent oxime-linked sugar linkers, first a medium scale reaction (≤0.250 mmol) was tested both in the presence and absence of 0.1 M aniline as a catalyst and equimolar quantities of sugar and linker. The aniline-based reaction conditions were superior to the reactions without aniline, which was not unexpected, however, if greener reaction conditions are sought or simplified purification procedures are desired, the reactions still work well without the catalyst when the reaction is carried out using microwave conditions. When the reaction was scaled up to a more preparative scale (≥0.800 mmol) in the presence of 0.1 M aniline, slightly lower yields were obtained, however, the yields were considered to be acceptable, given the ease of setup and the short reaction time needed to produce larger quantities of simple glycoconjugates.

The real advantage of using a microwave-mediated reaction in the formation of oxime-linked glycoconjugates was realized when the method was applied to one disaccharide aldose and two ketose sugars in a multivalent reaction. The complete substitution of multivalent glycoclusters can be difficult to achieve, as multiple, simultaneous reactions are required between the individual sugars and the reactive moieties on the multivalent scaffold. However, in this study using our optimized microwave-mediated reaction conditions developed with the monovalent sugar linkers, it was found that the tri-cellobiosyl product (**17**) was synthesized in a 94% yield, with no under-substitution products observed, while the tri-sialic acid (**20**) and trivalent di-sialic acid (**21**) were the predominant products formed in 82 and 88% yields, respectively. For the latter two reactions, the only other observed minor product in each case was the disubstituted glycocluster.

This newly developed microwave method allows for the efficient production of monovalent or multivalent glycoconjugates. It offers ease of set-up, consistent trial-to-trial reaction control through the use of programmable methods, short reaction times and good yields of the desired products, all while utilizing equimolar quantities of the reactant oxime-forming partners. This method, when applied to larger, more complex/hindered oligosaccharides gives rise to primarily the desired fully substituted products, with minimal to no production of undesired under-substituted products. This is valuable, particularly when one is working with sugars that are rare or expensive to produce/purchase.

## 4. Materials and Methods

### 4.1. General Methods

Unless otherwise noted all chemicals were purchased from commercial sources and used without further purification/treatment. All microwave reactions were carried out in a CEM MARS 5 microwave. All reaction solutions were freeze dried upon reaction completion prior to purification. Size exclusion chromatography (SEC) separations were conducted on either a BioRad BioLogic DuoFlow 10 system or a Pharmacia LC 500 system, using a BioRad 2.5 × 120 cm column packed with BioGel P-10 in 0.03 M NH_4_HCO_3_. 3.5 mL fractions were collected, and the absorbance measured at 214 nm and 225 nm. ^1^H and ^13^C spectra (internal methanol standard) in D_2_O were collected on a Bruker Avance III 500 MHz spectrometer. ^1^H NMR integration data for **17**, **20** and **21** were normalized to 1/3 of the total molecule. Mass spectrometry data were obtained at the Campus Chemical Instrument Center (CCIC) Mass Spectrometry and Proteomics Facility at The Ohio State University (OSU).

### 4.2. Synthesis of Monovalent Sugar-Linkers

#### 4.2.1. General Procedure for the Medium-Scale (0.187–0.250 mmol) Synthesis of Sugar-Linkers

##### Without Aniline-microwave or Traditional Heating

**Compounds 9-15** were synthesized using 1 equivalent of the aminooxy linker (**Compound 8**) [16], prepared as a 100 mg/mL solution in methanol. The appropriate volume of this solution was transferred to a flask and evaporated under reduced pressure, then freeze-dried to get an accurate mass of the oil. Next, 1 equivalent of: *N*-acetylglucosamine, cellobiose, gentiobiose, lactose, maltose, maltotriose or melibiose was separately added to each flask containing **Compound 8** [16]. These were each dissolved in 3.0 mL of 0.1 M ammonium acetate (NH_4_OAc) at a pH of 4.5. The reactions were conducted either stirring at 50 °C in an oil bath (traditional) or at 400 W in a microwave (CEM MARS 5) at 25% power with a 2 min ramp to temperature and a hold time of 30 min at a maximum temperature of 50 °C. After the reaction was complete, the solutions were freeze-dried. The products were then purified by flash chromatography in 6:4:0.5 CHCl_3_:MeOH:H_2_O, yielding off-white amorphous solids.

***Tert*-butyl *N*-[3-(2-{(E/Z)-[2-acetamido-2-deoxy-D-glucopyranosyl]oxime}ethoxy)propyl] carbamate** (**Compound 9**): 54.3 mg (0.232 mmol) of **Compound 8** plus 53.2 mg (0.241 mmol) of **Compound 1** were utilized, resulting in 68.9 mg (67.9%) of an off white solid (**Compound 9**). ^1^H NMR (500 MHz, D_2_O): ^1^H NMR (500 MHz, D_2_O): δ 7.49 (d, *J* = 6.2 Hz, 0.7H, E isomer), 6.83 (d, *J* = 6.6 Hz, 0.2H, Z isomer), 5.10, (t, *J* = 6.7 Hz, 0.2H, Z isomer), 4.67 (t, *J* = 6.8 Hz, 0.7H, E isomer), 4.32 (d, *J* = 9.8 Hz, 0.1 H, closed ring), 4.30-4.17 (m, overlapping, 2H), 4.17-4.03 (m, overlapping, 1H), 3.39-3.47 (m, overlapping, 9.2H), 3.41 (d, *J* = 3.4 Hz, 0.1H, closed ring), 3.10 (t, *J* = 6.3 Hz, 2H), 2.02 (s, 3H), 1.71 (p, *J* = 6.3, 13.0 Hz, 2H), 1.40 (s, 9H). ^13^C NMR (125 MHz, D_2_O with internal MeOH standard): δ 174.21, 171.20, 149.99, 148.78, 81.03, 72.79, 71.50, 71.07, 70.22, 69.43, 68.65, 68.60, 63.02, 52.07, 49.03, 37.20, 28.84, 23.42, 22.38, 22.14, 22.03. HRMS ESI+: Calc. for C_18_H_36_N_3_O_9_ (M + H)^+^: 438.2416. Found: 438.2455.

***Tert*-butyl *N*-[3-(2-{(E/Z)-[β-D-glucopyranosyl-(1→4)-D-glucopyranosyl]oxime}ethoxy)propyl] carbamate** (**Compound 10-microwave**): 43.7 mg (0.187 mmol) of **Compound 8** plus 63.9 mg (0.187 mmol) of **Compound 2** were utilized, resulting in 64.7 mg (63.1%) of an off white solid (**Compound 10**). ^1^H NMR (500 MHz, D_2_O): δ 7.69 (d, *J* = 5.5 Hz, 0.6H, E isomer), 7.00 (d, *J* = 5.5 Hz, 0.1H, Z isomer), 4.99 (dd, *J* = 4.1, 5.4 Hz, 0.1H, Z isomer), 4.58 (dd, *J* = 5.7, 6.8 Hz, 0.7H, E isomer), 4.56-4.49 (m, overlapping, 0.9H), 4.30 (d, *J* = 9.2 Hz, 0.2H), 4.28-4.21 (m, overlapping, 1.5H), 4.09 (t, *J* = 3.7 Hz, 0.1H, Z isomer), 3.98 (dd, *J* = 1.8, 6.9 Hz, 0.7H, E isomer), 3.96-3.39 (m, overlapping, 14.8 H), 3.46-3.29 (m, overlapping, 1H), 3.17-3.11 (m, overlapping, 2H), 1.75 (p, *J* = 6.5, 12.9 Hz, 2H), 1.43 (s, 9H). ^13^C NMR (125 MHz, D_2_O with internal MeOH standard): δ 158.39, 153.22, 152.06, 102.69, 90.25, 81.08, 80.77, 78.64, 78.38, 76.16, 75.97, 75.76, 75.45, 73.51. 73.35, 73.28, 72.81, 71.51, 71.34, 70.59, 69.63, 69.51, 69.52, 68.82, 68.66, 68.53, 68.49, 66.46, 62.27, 62.09, 60.76, 60.66, 60.33, 49.06, 37.29, 28.94, 27.88. HRMS ESI+: Calc. for C_22_H_43_N_2_O_14_ (M + H)^+^: 559.2709. Found: 559.2724.

**Cellobiose (Compound 10-oil bath, traditional):** 55.3 mg (0.236 mmol) of **Compound 8** plus 73.3 mg (0.214 mmol) of **Compound 2** were utilized, resulting in 67.3 mg (56.1%) of an off white solid (**Compound 10**).

***Tert*-butyl *N*-[3-(2-{(E/Z)-(β-D-glucopyranosyl-(1→6)-D-glucopyranosyl)oxime}ethoxy)propyl] carbamate** (**Compound 11**): 46.6 mg (0.199 mmol) of **Compound 8** plus 70.8 mg (0.207 mmol) of **Compound 3** were utilized, resulting in 65.8 mg (60.3%) of a fluffy white solid (**Compound 11**). ^1^H NMR (500 MHz, D_2_O): δ 7.49 (d, *J* = 6.5 Hz, 0.7H, E isomer), 6.84 (d, *J* = 6.3 Hz, 0.1H, Z isomer), 4.42 (d, *J* = 9.1 Hz, 1H), 4.39 (d, *J* = 3.7 Hz, 0.7H), 4.39 (t, *J* = 3.7 Hz, 2H), 3.88-3.36 (m, overlapping, 15H), 3.05 (t, *J* = 6.5 Hz, 2H), 1.67 (p, *J* = 6.5, 13.1 Hz, 2H), 1.34 (s, 9H). ^13^C NMR (125 MHz, D_2_O with internal MeOH standard): δ 157.48, 151.76, 150.70, 102.10, 89.60, 80.15, 75.94, 75.37, 75.22, 75.16, 74.90, 72.60, 72.47, 72.36, 72.30, 71.94, 70.72, 70.14, 69.80, 69.53, 69.48, 69.07, 69.01, 68.94, 68.66, 68.52, 67.90, 67.74, 67.63, 65.61, 59.99, 48.17, 36.35, 28.02, 27.00, 22.53. HRMS ESI+: Calc. for C_22_H_43_N_2_O_14_ (M + H)^+^: 559.2715. Found: 559.2723.

***Tert*-butyl *N*-[3-(2-{(E/Z)-(β-D-galactopyranosyl-(1→4)-D-glucopyranosyl)oxime}ethoxy)propyl] carbamate** (**Compound 12**): 45.0 mg (0.192 mmol) of **Compound 8** plus 65.8 mg (0.192 mmol) of **Compound 4** were utilized, resulting in 68.1 mg (64.6%) of an off white solid (**Compound 12**). ^1^H NMR (500 MHz, D_2_O): δ 7.70 (d, *J* = 5.5 Hz, 0.7H, E isomer), 7.00 (d, *J* = 5.4 Hz, 0.1H, Z isomer), 4.59 (d, *J* = 6.0 Hz, 0.7H), 4.50 (d, *J* = 7.8 Hz, 0.8H), 4.24 (t, *J* = 4.4 Hz, 2H), 3.98-3.54 (m, overlapping, 16H), 3.14 (t, *J* = 6.4 Hz, 2H), 1.76 (p, *J* = 6.5, 13.0 Hz, 2H), 1.43 (s, 9H). ^13^C NMR (125 MHz, D_2_O with internal MeOH standard): δ 158.43, 153.38, 152.23, 103.70, 103.26, 103.13, 90.30, 81.08, 80.86, 78.50, 78.35, 76.23, 75.56, 75.43, 75.27, 73.58, 73.32, 72.85, 72.76, 71.52, 71.39, 71.26, 71.18, 70.55, 69.60, 69.48, 68.89, 68.79, 68.71, 68.54, 66.75, 62.31, 62.11, 61.26, 61.09, 61.00, 60.39, 49.12, 37.29, 28.99, 27.95. HRMS ESI+: Calc. for C_22_H_43_N_2_O_14_ (M + H)^+^: 559.2715. Found: 559.2727.

***Tert*-butyl *N*-[3-(2-{(E/Z)-(α-D-glucopyranosyl-(1→4)-D-glucopyranosyl)oxime}ethoxy)propyl] carbamate** (**Compound 13**): 52.0 mg (0.222 mmol) of **Compound 8** plus 80.5 mg (0.223 mmol) of **Compound 5** were utilized, resulting in 79.5 mg (65.3%) of a white powdery solid (**Compound 13**). ^1^H NMR (500 MHz, D_2_O): δ 7.51 (d, *J* = 6.1 Hz, 0.7H, E isomer), 6.88 (d, *J* = 5.5 Hz, 0.2H, Z isomer), 5.02 (d, *J* = 4.0 Hz, 1H), 4.44 (t, *J* = 5.5 Hz, 0.7H), 4.16 (t, *J* = 1.7 Hz, 2H), 4.92-3.35 (m, overlapping, 16H), 3.04 (t, *J* = 6.6 Hz, 2H), 1.67 (p, *J* = 6.5, 13.1 Hz, 2H), 1.35 (s, 9H). ^13^C NMR (125 MHz, D_2_O with internal MeOH standard): δ 158.38, 153.53, 151.85, 129.60, 128.03, 125.53, 100.73, 100.59, 99.83, 90.27, 81.61, 81.04, 80.34, 77.36, 76.87, 75.94, 73.52, 73.27, 73.12, 73.03, 72.94, 72.86, 72.64, 72.42, 72.29, 71.84, 71.45, 69.59, 69.54, 69.49, 69.43, 68.83, 68.68, 68.66, 68.48, 65.86, 62.48, 62.26, 60.98, 60.64, 60.54, 49.05, 37.27, 28.91, 27.89. HRMS ESI+: Calc. for C_22_H_43_N_2_O_14_ (M + H)^+^: 559.2715. Found: 559.2713.

***Tert*-butyl *N*-[3-(2-{(E/Z)-(α-D-glucopyranosyl-(1→4)-α-D-glucopyranosyl-(1→4)-D-glucopyranosyl)oxime} ethoxy)propyl] carbamate** (**Compound 14**): 53.4 mg (0.228 mmol) of **Compound 8** plus 115.5 mg (0.229 mmol) of **Compound 6** were utilized, resulting in 112.2 mg (68.3%) of an off white amorphous solid (**Compound 14**). ^1^H NMR (500 MHz, D_2_O): δ 7.62 (d, *J* = 6.1 Hz, 0.7 H, E isomer), 6.96 (d, *J* = 5.5 Hz, 0.1 H, Z isomer), 5.59 (d, *J* = 3.9 Hz, 1 H), 5.36 (d, *J* = 4.0 Hz, 0.8 H), 4.54 (t, *J* = 5.6 Hz, 0.7 H), 4.29 (t, *J* = 3.7 Hz, 2 H), 4.04-3.61 (m, overlapping, 21H), 3.58 (t, *J* = 3.5 Hz, 1 H), 3.15 (t, *J* = 6.3 Hz, 2H), 1.77 (p, *J* = 6.5, 13.0 Hz, 2H), 1.45 (s, 9H). ^13^C NMR (125 MHz, D_2_O with internal MeOH standard): δ 158.51, 153.68, 151.96, 100.53, 100.06, 81.84, 81.14, 80.54, 77.46, 77.11, 76.04, 73.69, 73.65, 73.52, 73.20, 72.99, 72.94, 72.51, 72.44, 72.08, 71.77, 71.62, 71.51, 71.30, 69.72, 69.62, 69.52, 68.96, 68.79, 68.60, 65.99, 62.57, 62.36, 61.03, 60.77, 60.65, 49.15, 37.37, 29.04, 27.99. HRMS ESI+: Calc. for C_28_H_53_N_2_O_19_ (M + H)^+^: 721.3244. Found: 721.3244.

***Tert*-butyl *N*-[3-(2-{(E/Z)-(α-D-galactopyranosyl-(1→6)-α-D-glucopyranosyl-(1→4)-D-glucopyranosyl)oxime}ethoxy)propyl] carbamate** (**Compound 15**): 46.1 mg (0.197 mmol) of **Compound 8** plus 70.9 mg (0.197 mmol) of **Compound 7** were utilized, resulting in 73.0 mg (67.6%) of an off white amorphous solid (**Compound 15**). ^1^H NMR (500 MHz, D_2_O): δ 7.60 (d, *J* = 6.5 Hz, 0.7H, E isomer), 6.95 (d, *J* = 9.4 Hz, 0.1H, Z isomer), 4.99 (d, *J* = 3.6 Hz, 2H), 4.44 (d, *J* = 6.8 Hz, 0.7H), 4.26 (d, *J* = 6.8 Hz, 2H), 4.00-3.51 (m, overlapping, 16H), 1.75 (p, *J* = 6.6, 13.1 Hz, 2H), 1.44 (s, 9H). ^13^C NMR (125 MHz, D_2_O with internal MeOH standard): δ 158.44, 152.85, 151.76, 98.58, 81.11, 75.97, 73.27, 72.92, 71.17, 71.12, 71.04, 70.61, 70.49, 70.36, 69.99, 69.77, 69.52, 69.48, 68.94, 68.88, 68.84, 68.72, 68.70, 66.67, 61.37, 61.31, 57.66, 49.12, 37.31, 28.98, 27.96, 22.39, 17.04. HRMS ESI+: Calc. for C_22_H_42_N_2_NaO_14_ (M + Na)^+^: 581.2535. Found: 581.2556.

##### With Aniline

**Compounds 9-15** were synthesized using 1 equivalent of the aminooxy linker (**Compound 8**) [16], prepared as a 100 mg/mL solution in methanol. The appropriate volume of this solution was transferred to a flask and evaporated under reduced pressure, then freeze-dried to get an accurate mass of the oil. Next, 1 equivalent of: *N*-acetylglucosamine, cellobiose, gentiobiose, lactose, maltose, maltotriose or melibiose was separately added to each flask containing **Compound 8**. These were each dissolved in 3.0 mL of 0.1 M ammonium acetate (NH_4_OAc) at a pH of 4.5. Next, 27.3 µL of aniline (0.1 M final concentration) were added to each flask and the pH checked to ensure that it remained at 4.5. The reactions were conducted either stirring at 50 °C in an oil bath (traditional) or at 400 W in a microwave (CEM MARS 5) at 25% power with a 2 min ramp to temperature and a hold time of 30 min at a maximum temperature of 50 °C. After the reaction was complete, the solutions were freeze-dried. The products were then purified by flash chromatography in 6:4:0.5 CHCl_3_:MeOH:H_2_O, yielding off white amorphous solids.

***N*-acetylglucosamine (Compound 9):** 55.3 mg (0.240 mmol) of **Compound 8** plus 52.2 mg (0.240 mmol) of **Compound 1** were utilized, resulting in 81.4 mg (77.6%) of an off white solid (**Compound 9**).

**Cellobiose (Compound 10-microwave):** 53.1 mg (0.227 mmol) of **Compound 8** plus 77.7 mg (0.227 mmol) of **Compound 2** were utilized, resulting in 96.3 mg (76.1%) of an off white solid (**Compound 10**).

**Cellobiose (Compound 10-oil bath, traditional):** 50.4 mg (0.215 mmol) of **Compound 8** plus 74.2 mg (0.217 mmol) of **Compound 2** were utilized, resulting in 77.8 mg (64.8%) of an off white solid (**Compound 10**).

**Gentiobiose (Compound 11):** 52.0 mg (0.222 mmol) of **Compound 8** plus 80.1 mg (0.222 mmol) of **Compound 3** were utilized, resulting in 90.6 mg (73.1%) of a fluffy white solid (**Compound 11**).

**Lactose (Compound 12):** 56.5 mg (0.241 mmol) of **Compound 8** plus 82.6 mg (0.241 mmol) of **Compound 4** were utilized, resulting in 124.7 mg (92.7%) of an off white solid (**Compound 12**).

**Maltose (Compound 13):** 43.6 mg (0.186 mmol) of **Compound 8** plus 67.0 mg (0.186 mmol) of **Compound 5** were utilized, resulting in 76.4 mg (73.6%) of an off white amorphous solid (**Compound 13**).

**Maltotriose (Compound 14):** 48.1 mg (0.206 mmol) of **Compound 8** plus 103.7 mg (0.206 mmol) of **Compound 6** were utilized, resulting in 137.0 mg (92.3%) of an off white amorphous solid (**Compound 14**).

**Melibiose (Compound 15):** 47.7 mg (0.204 mmol) of **Compound 8** plus 73.4 mg (0.204 mmol) of **Compound 7** were utilized, resulting in 91.2 mg (80.1%) of an off white amorphous solid (**Compound 15**).

#### 4.2.2. General Procedure for the Large-scale (≥0.800 mmol) Synthesis of Sugar-linkers:

**Compounds 9-15** were synthesized using 1 equivalent of the aminooxy linker (**Compound 8)**, [16] prepared as a 100 mg/mL solution in methanol. The appropriate volume of this solution was transferred to a flask and evaporated under reduced pressure, then freeze-dried to get an accurate mass. Next, 1 equivalent of: *N*-acetylglucosamine, cellobiose, gentiobiose, lactose, maltose, maltotriose or melibiose was separately added to each flask containing **Compound 8**. These were dissolved in 5.0 mL of 0.1 M ammonium acetate (NH_4_OAc) at a pH of 4.5, followed by 45.6 µL of aniline (0.1 M final concentration). The pH was checked after aniline addition to confirm it was still 4.5. The reactions were conducted at 400 W in a microwave (CEM MARS 5) at 25% power with a 2 min ramp to temperature and a hold time of 30 min at a maximum temperature of 50 °C. After the reaction was complete, the solutions were freeze-dried. The products were then purified by flash chromatography in 6:4:0.5 CHCl_3_:MeOH:H_2_O followed by dialysis in 100 molecular weight cutoff (MWCO) tubing against water (for gentiobiose, maltose and maltotriose only), yielding white to off-white solids.

***N*-acetylglucosamine (Compound 9):** 213.5 mg (0.912 mmol) of **Compound 8** plus 201.6 mg (0.912 mmol) of **Compound 1** were utilized, resulting in 315.8 mg (79.2%) of a white amorphous solid (**Compound 9**).

**Cellobiose (Compound 10):** 186.8 mg (0.798 mmol) of **Compound 8** plus 273.0 mg (0.798 mmol) of **Compound 2** were utilized, resulting in 333.6 mg (74.9%) of a white solid (**Compound 10**).

**Gentiobiose (Compound 11):** 205.1 mg (0.876 mmol) of **Compound 8** plus 299.8 mg (0.876 mmol) of **Compound 3** were utilized, resulting in 319.4 mg (65.3%) of an off-white solid (**Compound 11**).

**Lactose (Compound 12):** 196.8 mg (0.841 mmol) of **Compound 8** plus 303.0 mg (0.841 mmol) of **Compound 4** were utilized, resulting in 293.5 mg (62.5%) of an off-white solid (**Compound 12**).

**Maltose (Compound 13):** 203.2 mg (0.868 mmol) of **Compound 8** plus 312.9 mg (0.868 mmol) of **Compound 5** were utilized, resulting in 299.8 mg (61.9%) of a white powdery solid (**Compound 13**).

**Maltotriose (Compound 14):** 208.0 mg (0.889 mmol) of **Compound 8** plus 448.4 mg (0.889 mmol) of **Compound 6** were utilized, resulting in 412.4 mg (64.3%) of an off-white solid (**Compound 14**).

**Melibiose (Compound 15):** 193.8 mg (0.828 mmol) of **Compound 8** plus 298.4 mg (0.828 mmol) of **Compound 7** were utilized, resulting in 275.9 mg (59.7%) of an off-white powdery solid (**Compound 15**).

### 4.3. Synthesis of Trivalent Glycoclusters

**(Cellobiose)_3_-Glycocluster, Compound 17: Compound 16** [16] was transferred to a round-bottomed flask as a 10 mg/mL solution in methanol and then evaporated under reduced pressure to give 9.9 mg (0.0183 mmol) of **Compound 16** as an oil. Next, 3 equivalents of **Compound 2** (18.8 mg, 0.0549 mmol) were added to the reaction flask. The solutes were dissolved in 1.5 mL of 0.1 M NH_4_OAc buffer, pH 4.5, plus 13.7 µL aniline (0.1 M final concentration) as a catalyst. The pH was confirmed to be 4.5 after aniline addition. The reaction was conducted at 400 W in a microwave (CEM MARS 5) at 25% power for 30 min. After the reaction was complete, the solution was freeze-dried then purified by SEC as described in general methods, yielding **Compound 17** (26 mg, 93.9%) as an off white solid. ^1^H NMR (500 MHz, D_2_O): δ 7.67 (d, *J* = 5.5 Hz, 0.5 H, E isomer), 7.00 (d, *J* = 5.5 Hz, 0.1H, Z isomer), 4.61–4.51 (m, 1.4H), 4.31–4.08 (m, 2H), 3.99–3.89 (m, 4H), 3.87-3.66 (m, 7H), 3.65–3.50 (m, 1H), 3.49–3.39 (m, 3H), 2.70 (br s, 2H), 2.53 (app t, 2H). ^13^C NMR (125 MHz, D_2_O with internal MeOH standard): δ 174.39, 152.17, 102.83, 90.39, 78.72, 78.48, 76.30, 76.10, 75.87, 75.81, 75.58, 73.63, 73.59, 73.47, 72.92, 71.42, 69.76, 69.70, 69.63, 69.02, 68.81, 67.09, 66.97, 62.37, 60.89, 60.77, 52.70, 36.27. MALDI-MS: Calc for C_57_H_106_N_7_O_39_ (M + H)^+^: 1512.652. Found: 1512.687.

**(Sialic Acid)_3_-Glycocluster, Compound 20: Compound 16** [16] was transferred to a pear shaped flask as a 10 mg/mL solution in methanol, then evaporated, yielding 10.8 mg (0.02 mmol) of the trivalent core. Next, 3 equivalents of sialic acid (**Compound 18**, Nacalai Tesque, 18.6 mg, 0.06 mmol) were weighed into the flask. The solutes were then dissolved in 1.5 mL of 0.1 M NH_4_OAc, pH 4.5, plus 13.7 µL aniline (0.1 M final concentration) as a catalyst. The pH was confirmed to be 4.5 after aniline addition. The reaction was conducted at 400 W in a microwave (CEM MARS 5) at 25% power for 30 min. After the reaction was complete, the solution was freeze-dried then purified by SEC as described in general methods, yielding **Compound 20** (23 mg, 81.6%) of an off white solid. ^1^H NMR (500 MHz, D_2_O): δ 4.41 (m, 1H), 4.28 (app t, 1.5H), 4.15 (app t, 0.5H), 4.01-3.91 (m, 2H), 3.85-3.73 (m, 6H), 3.65-3.56 (m, 3H), 3.47-3.45 (app d, 1H), 3.39-3.36 (m, 2H), 2.82-2.70 (m, 1.5H), 2.58 (app t, 2H), 2.47 (d, *J* = 6.8 Hz, 0.5H), 2.08 (s, 3H). ^13^C NMR (125 MHz, D_2_O with internal MeOH standard): δ 175.10, 175.07, 174.50, 170.44, 170.05, 157.22, 156.15, 73.33, 72.65, 70.72, 69.50, 69.45, 69.02, 68.90, 67.90, 67.86, 66.71, 66.55, 65.97, 63.35, 53.94, 53.56, 53.49, 53.39, 35.90, 35.83, 34.70, 34.64, 30.94, 22.05, 22.01. MALDI-MS: Calc. for C_54_H_96_N_10_O_33_ (M + H)^+^: 1413.62134. Found: 1413.799.

**(Disialic Acid)_3_-Glycocluster, Compound 21: Compound 16** [16] was transferred to a pear shaped flask as a 10 mg/mL solution in methanol, then evaporated, yielding 5.9 mg (0.011 mmol) of the trivalent core as an oil. Next, the α-2→8 linked dimer of sialic acid (**Compound 19**, disialic acid, 21.1 mg, 0.033 mmol) [35] was added. The solutes were then dissolved in 1.5 mL of 0.1 M NH_4_OAc, pH 4.5, plus 13.7 µL of aniline (0.1 M final concentration) as a catalyst. The pH was confirmed to be 4.5 after aniline addition. The reaction was conducted at 400 W in a microwave (CEM MARS 5) at 25% power for 90 min with a temperature maximum set at 50 °C. After the reaction was complete, the solution was freeze-dried then purified by FPLC as described in general methods, yielding **Compound 21** (22 mg, 87.6%) as an off white solid. ^1^H NMR (500 MHz, D_2_O): δ 4.46-4.40 (m, 1H), 4.29 (app t, 1.5H), 4.15 (app t, 0.5H), 3.99-3.91 (m, 4H), 3.89-3.74 (m, 9.5H), 3.70-3.61 (m, 4H), 3.57-3.53 (m, 3H), 3.26 (br s, 2H), 2.83-2.67 (m, 2.5H), 2.57 (t, *J* = 5.9 Hz, 2H), 2.47 (m, 0.5H), 2.09 (s, 3H), 2.03 (s, 3H), 1.78 (t, *J* = 12.2 Hz, 1H). ^13^C NMR (125 MHz, D_2_O with internal MeOH standard): δ 175.20, 174.89, 174.36, 173.47, 101.84, 74.30, 74.24, 72.71, 72.51, 71.80, 71.73, 68.96, 68.77, 68.21, 68.05, 67.75, 67.70, 67.49, 67.45, 66.57, 66.28, 65.79, 62.66, 61.14, 53.72, 53.40, 53.36, 53.31, 51.65, 40.05, 35.79, 35.75, 35.68, 34.42, 30.82, 21.97, 21.95. MALDI-MS: Calc. for C_87_H_147_N_13_O_57_ (M + H)^+^: 2286.90755. Found: 2286.791.

## Figures and Tables

**Table 1:** Summary of sugar-linker 50 °C traditional and microwave syntheses. Medium scale (≤0.250 mmol) reactions were run in the presence or absence of 0.1 M aniline (final concentration), while the large scale (≥0.800 mmol) reactions were all run in the presence of 0.1 M aniline as a catalyst. The Δ% yield column compares the yields of the medium scale aniline catalyzed reaction with the uncatalyzed reaction of the same scale. For the large-scale reactions, the comparison is between the medium and large-scale aniline-catalyzed reactions.

Sugar	Additive	% Yield	% Yield
**Medium Scale (≤0.250 mmol)**			
**Non-Microwave Conditions:**			
Cellobiose	0.1 M Aniline	65	
	N/A	56	−9
**Microwave-Mediated Conditions:**			
GlcNAc	0.1 M Aniline	78	
	N/A	68	−10
Cellobiose	0.1 M Aniline	76	
	N/A	63	−13
Gentiobiose	0.1 M Aniline	73	
	N/A	60	−13
Lactose	0.1 M Aniline	93	
	N/A	65	−28
Maltose	0.1 M Aniline	74	
	N/A	65	−9
Maltotriose	0.1 M Aniline	92	
	N/A	68	−24
Melibiose	0.1 M Aniline	80	
	N/A	68	−12
**Large Scale (≥0.800 mmol)**			
GlcNAc	0.1 M Aniline	79	1
Cellobiose	0.1 M Aniline	75	−1
Gentiobiose	0.1 M Aniline	65	−8
Lactose	0.1 M Aniline	63	−30
Maltose	0.1 M Aniline	62	−12
Maltotriose	0.1 M Aniline	64	−28
Melibiose	0.1 M Aniline	60	−20

## Supplementary Materials

The following are available online at https://www.mdpi.com/1424-8247/12/1/39/s1, Figures S1–S20: ^1^H (odd) and ^13^C (even) NMR spectra, Table S1: Summary of the E/Z ratios and % ring open oxime for all products.
