# Supplementary material for: Development of a Microwave-assisted Chemoselective Synthesis of Oxime-linked Sugar Linkers and Trivalent Glycoclusters
Source: Pharmaceuticals (Basel). 2019 Mar 14;12(1):39. doi: 10.3390/ph12010039 (PMC6469176; doi:10.3390/ph12010039)
Supplement: Supplementary file 1 [file pharmaceuticals-12-00039-s001.pdf]

Supplementary Materials

Development of a Microwave-assisted Chemoselective Synthesis of Oxime-linked Sugar Linkers and Trivalent Glycoclusters

Katherine McReynolds [1,*], Dustin Dimas [1,2], Grace Floyd [1,3] and Kara Zeman [1]

[1] Department of Chemistry, California State University, Sacramento, 6000 J Street, Sacramento, CA 95819-6057, USA; Dustin.Dimas-1@ou.edu; gracepfloyd@gmail.com; karazeman@csus.edu;

[2] Current address: Department of Chemistry and Biochemistry, The University of Oklahoma, 101 Stephenson Parkway, SLSRC, Room 1000, Norman, OK 73019-5251, USA

[3] Current address: Biocare Medical, 60 Berry Dr., Pacheco, CA 94553, USA

* Correspondence: kdmcr@csus.edu; Tel.: +01-916-278-6551

Table of Contents **Page**

1. NMR Analysis 2

4. ^{1}H and ^{13}C spectra 4

5. References 24

NMR Analysis:

In protic solutions such as D_2O, the oxime products reported in this paper would consist of a mixture of not only the ring opened E/Z isomers (major), but also the ring closed β-glycosides (minor) as illustrated in **Figure 1** of the main body of the paper.[2] Given the persistence of these different isomers, it is important to note some of the features present in the ^1H NMR spectra.

First, in the ^1H spectra of **9-15** and **17**, the two downfield signals found between 7.0-8.0 ppm correspond to the open chain E/Z isomers, with the most downfield and dominant signal the E isomer, and the more upfield signal the Z isomer. **Table S1** summarizes the E/Z ratios for the products. What can also be noted from the ^1H NMR spectra of **9-15** and **17** is the presence of several small signals integrating for less than 1H each. These should not be interpreted as impurities, but instead as minor isomer species. All of the products correspond to either a single spot by TLC or a single peak eluted by SEC. Additionally, the products all give rise to the expected number of protons, and finally, they have all been confirmed by high-resolution mass spectrometry (HRMS). For example, from the evaluation of the ^1H NMR of **Compound 9**, it can clearly be seen that the expected ratio of 3:1 for the Boc on the linker (1.40 ppm) to the acetyl protons on the sugar (2.02 ppm) matches that calculated using the peak integrations, despite the sum of the oxime signals at 7.49 ppm (E isomer) and 6.83 ppm (Z isomer) adding to only 0.9 total protons. Based on this information, it is therefore reasonable that the integration of the combined ring opened oxime E and Z signals will not integrate to a full proton, but that some portion of the product is present in the ring-closed form. It would be expected that most of the ring-closed glycosides would predominantly exist in the more favorable β-configuration where the linker is equatorial. A determination of the α/β ratio of these minor products could not be obtained from the current 1D ^1H NMR data given the complexity of the signals overlapping in the region where the anomeric protons would be observed, and the expectation that the α-isomer would only be

present in trace amounts at the most. However, an estimation of the percentage of product in the ring open oxime could be made from the sum of the E and Z integrations (**Table S1**) and was found to range from 71-90%, depending on the sugar.

Table S1: Summary of the E/Z ratios and % ring open oxime for all products. For all but **20** and **21**, this ratio was determined from the ratio of the E and Z proton signals found between 7.0-8.0 ppm in the ^{1}H spectra. For **20** and **21**, this number was estimated from signals found at 4.15 and 4.28-4.29 ppm, which correspond to the first methylene of the core molecule adjacent to the oxime.[1] ND: Not determined.

Sugar (Compound #)	E/Z Ratio	% Ring open oxime
**Monovalent**		
GlcNAc (9)	3.5:1	90
Cellobiose (10)	5.5:1	71
Gentiobiose (11)	4.4:1	87
Lactose (12)	7.1:1	81
Maltose (13)	4.4:1	74
Maltotriose (14)	4.6:1	79
Melibiose (15)	5.4:1	83
**Trivalent**		
Cellobiose (17)	5.4:1	60
Sialic Acid (20)	3.0:1	ND
Disialic Acid (21)	2.7:1	ND

For the ketose-derived products, **20** and **21**, there is no oxime proton present, therefore, no signals appear in the 7.0-8.0 ppm range as was seen for the aldose-derived products, **9-15** and **17**. Instead, a strong signal corresponding to the proximal methylene group of the core adjacent to the oxime was used. This group gives rise to two broad apparent triplet signals at 4.28-29 (E isomer) and 4.15 ppm (Z isomer) for **20** and **21**. These signals were assigned based on their similarity to the linker molecule used in the NMR study reported by Szabo and coworkers.[1] The ratio of these two peaks was therefore used to estimate the E/Z ratio of the ring-opened oxime present. Unfortunately, as there is no E/Z proton available, the percentage of ring-opened oxime could not be estimated for **20** and **21.**

Figure S1: ^{1}H NMR of **Compound 9** (500 MHz, D_2O).

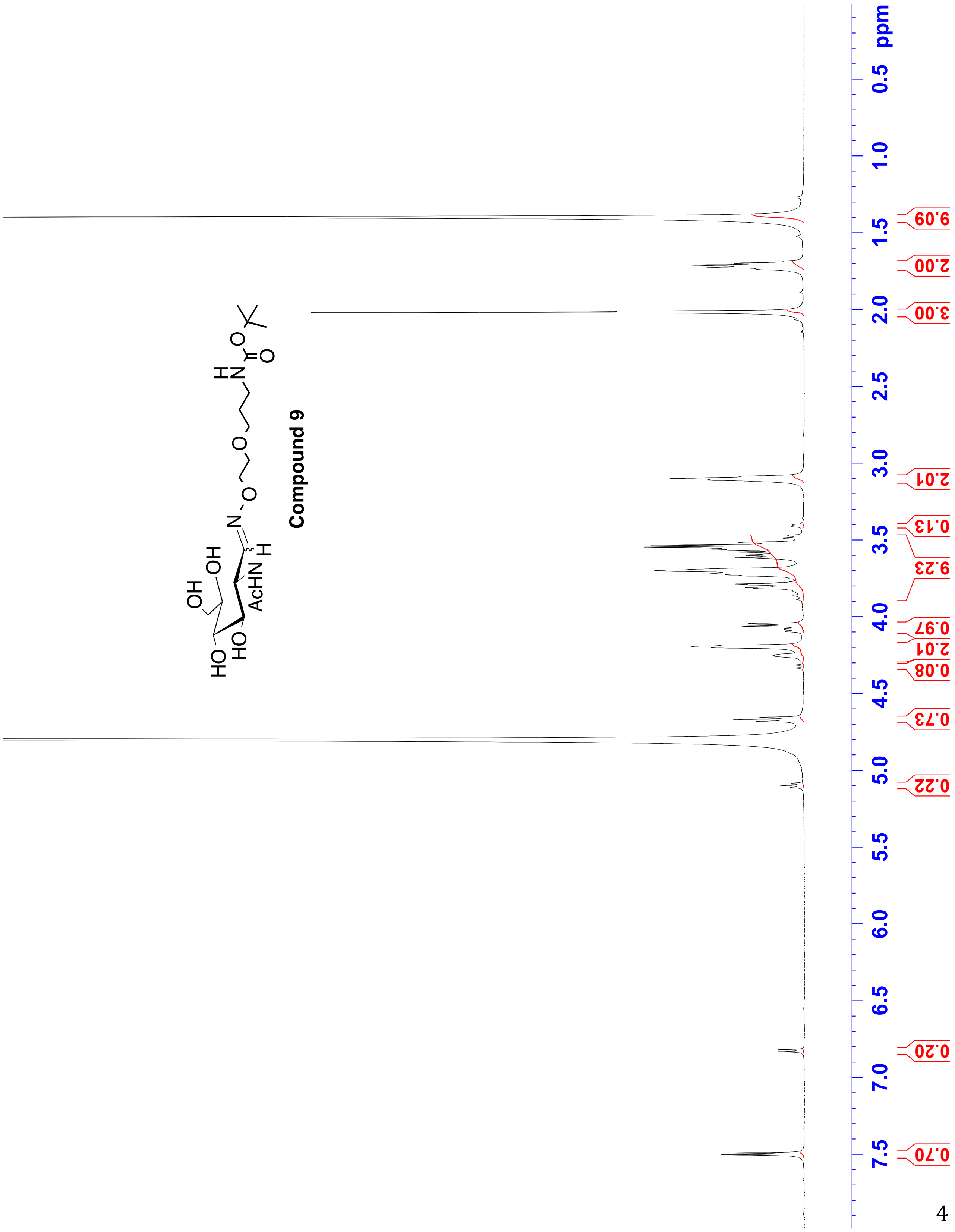

Figure S2: ^{13}C NMR of **Compound 9** (125 MHz, D_2O, internal MeOH standard).

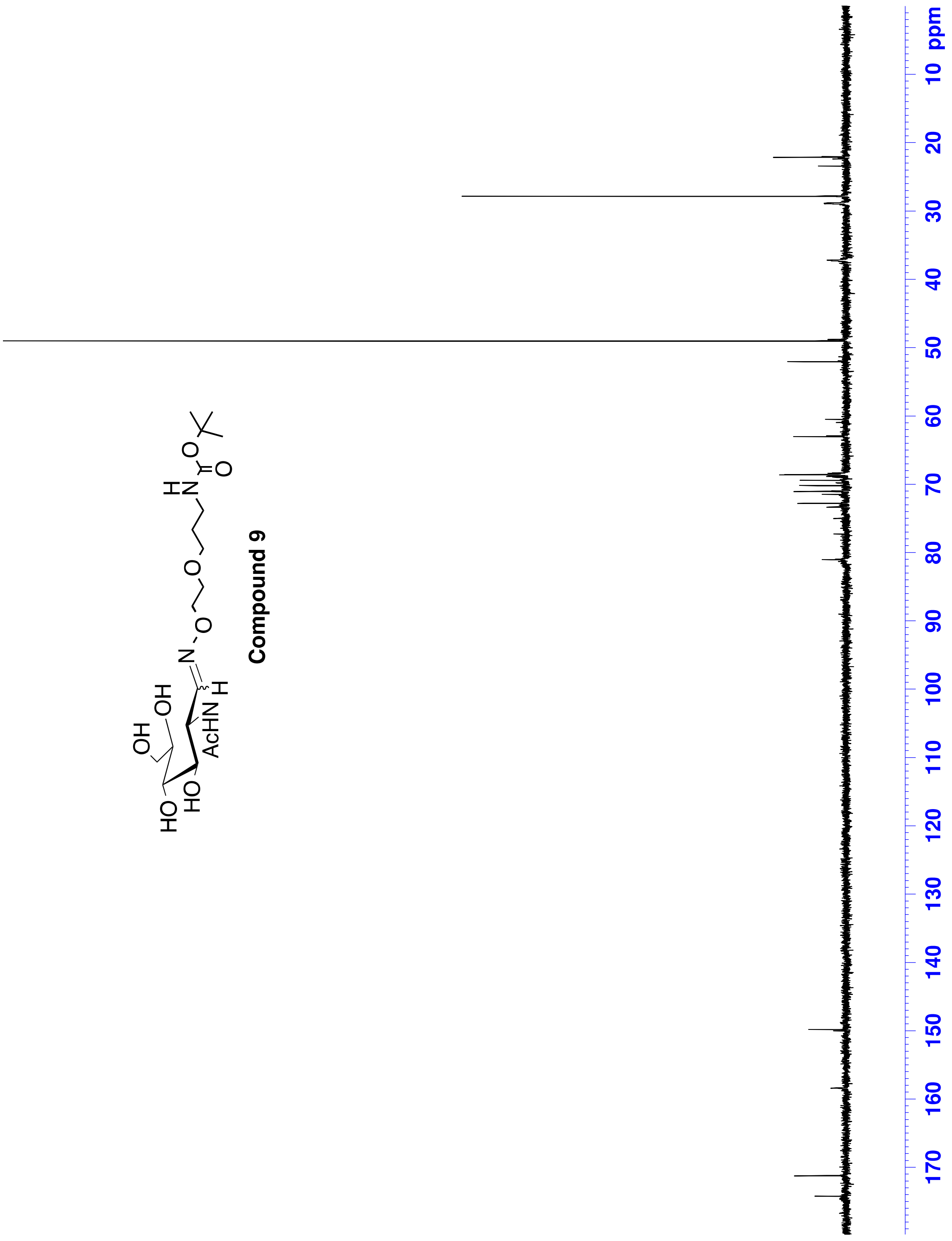

Figure S3: ^{1}H NMR of **Compound 10** (500 MHz, D_2O).

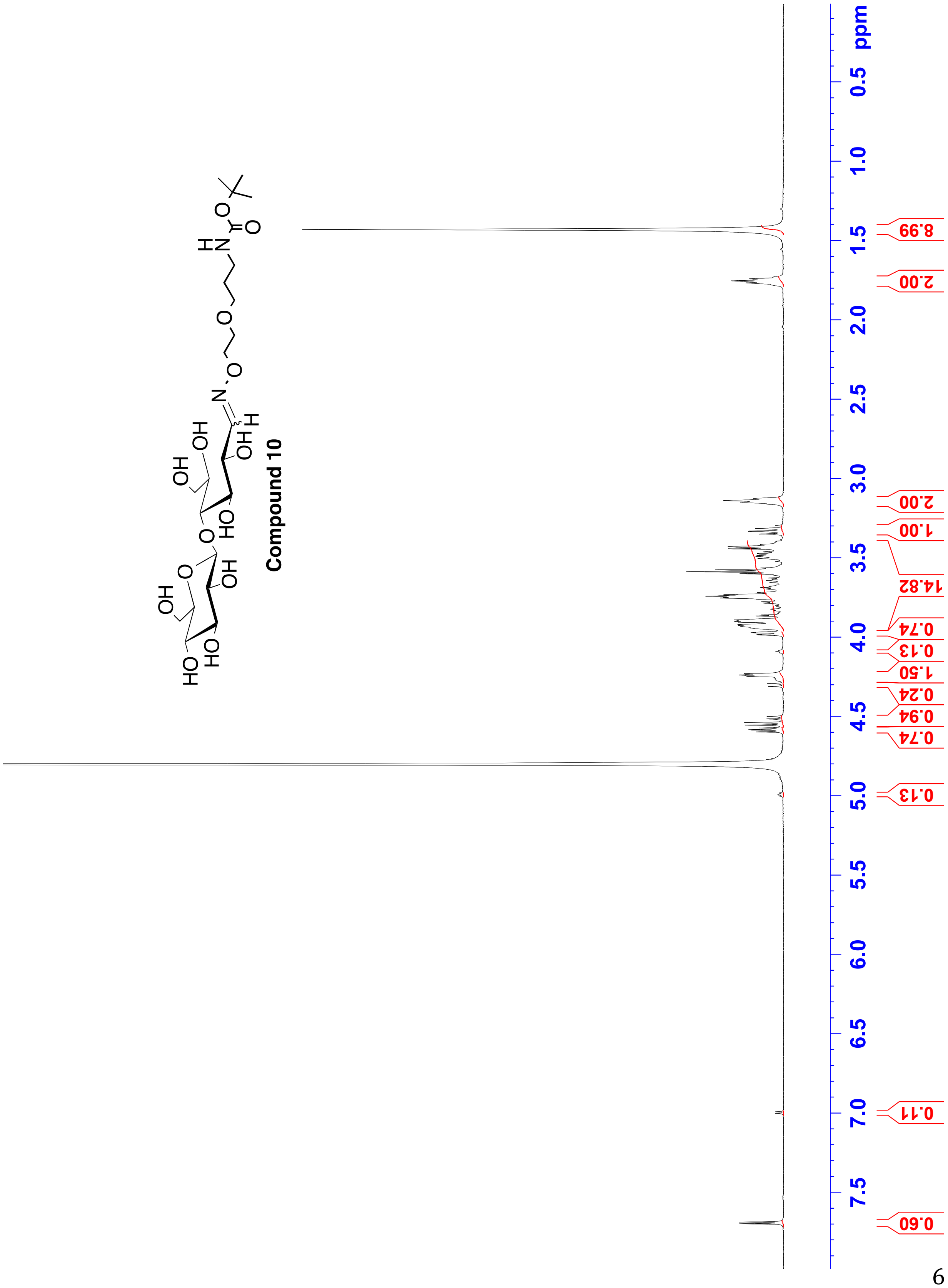

Figure S4: ^{13}C NMR of **Compound 10** (125 MHz, D_2O, internal MeOH standard).

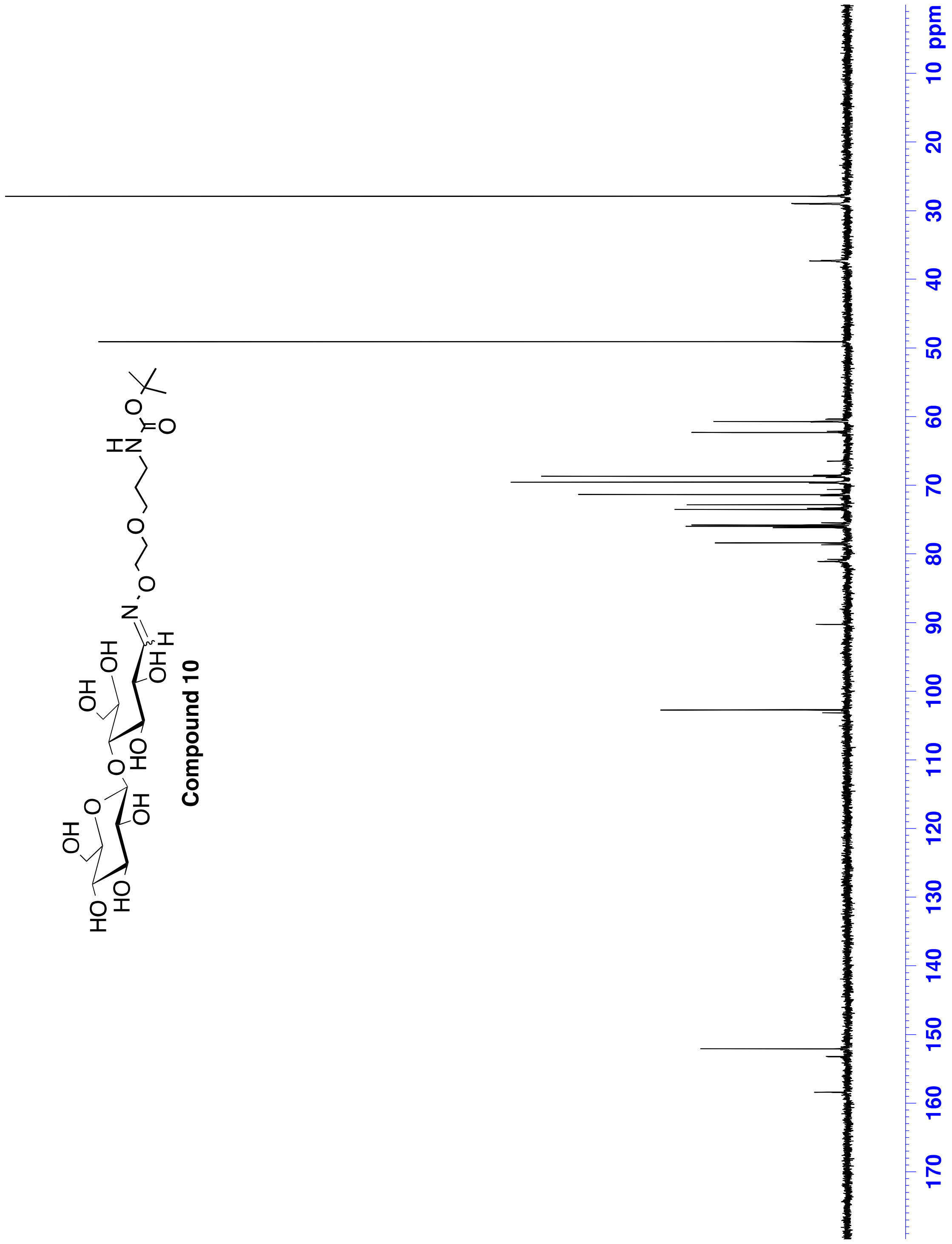

Figure S5: ^{1}H NMR of **Compound 11** (500 MHz, D_2O).

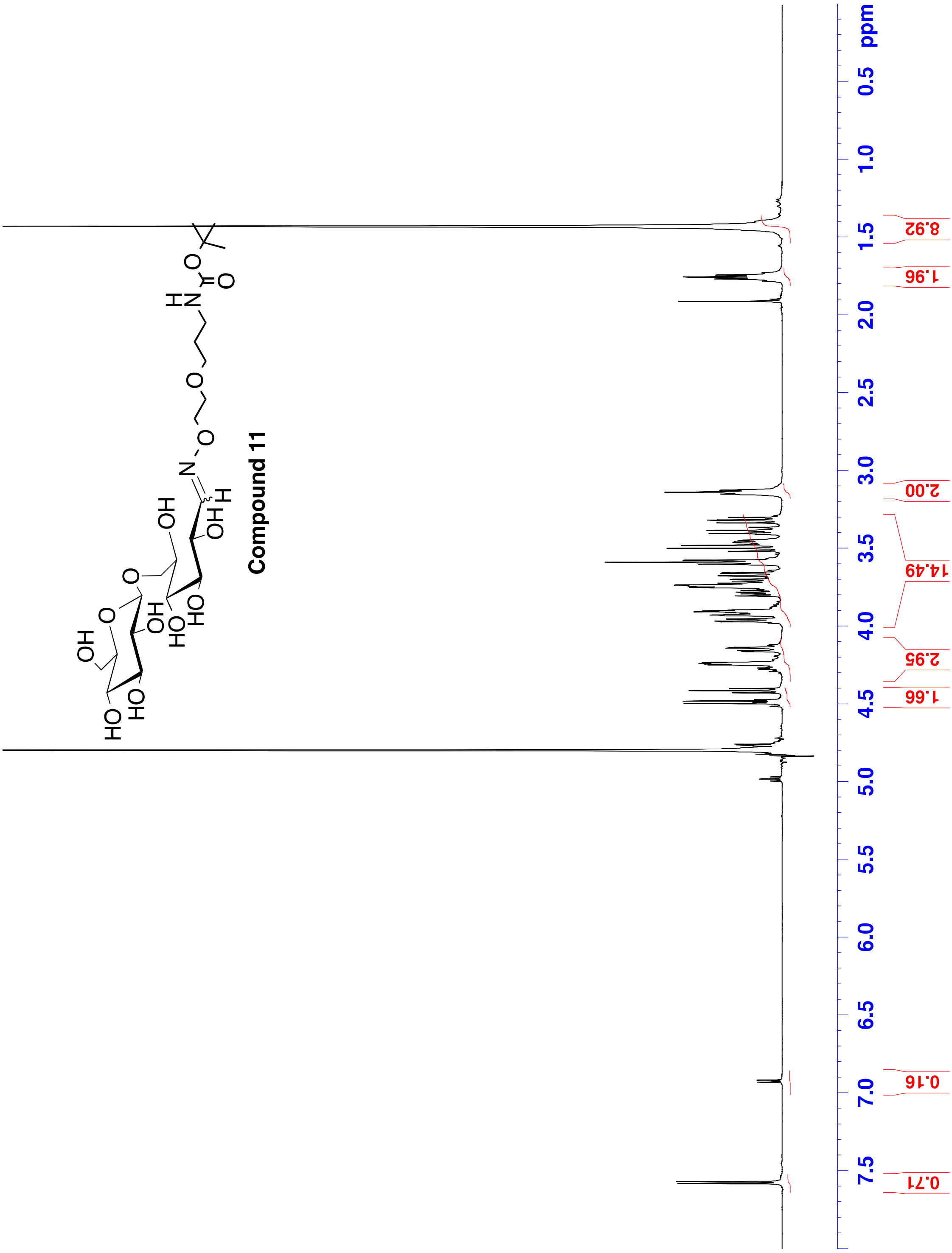

Figure S6: ^{13}C NMR of **Compound 11** (125 MHz, D_2O, internal MeOH standard).

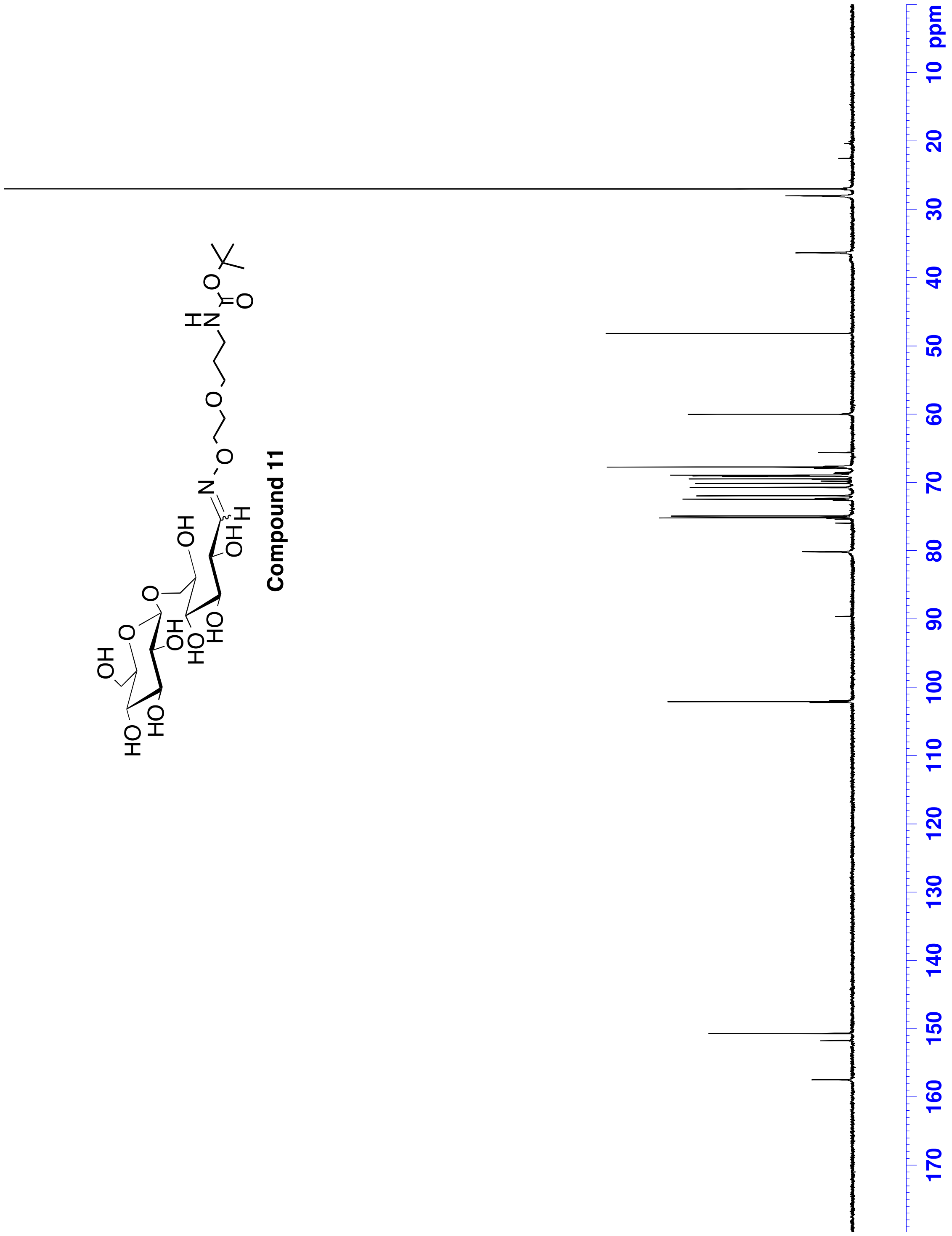

Figure S7: ^{1}H NMR of **Compound 12** (500 MHz, D_2O).

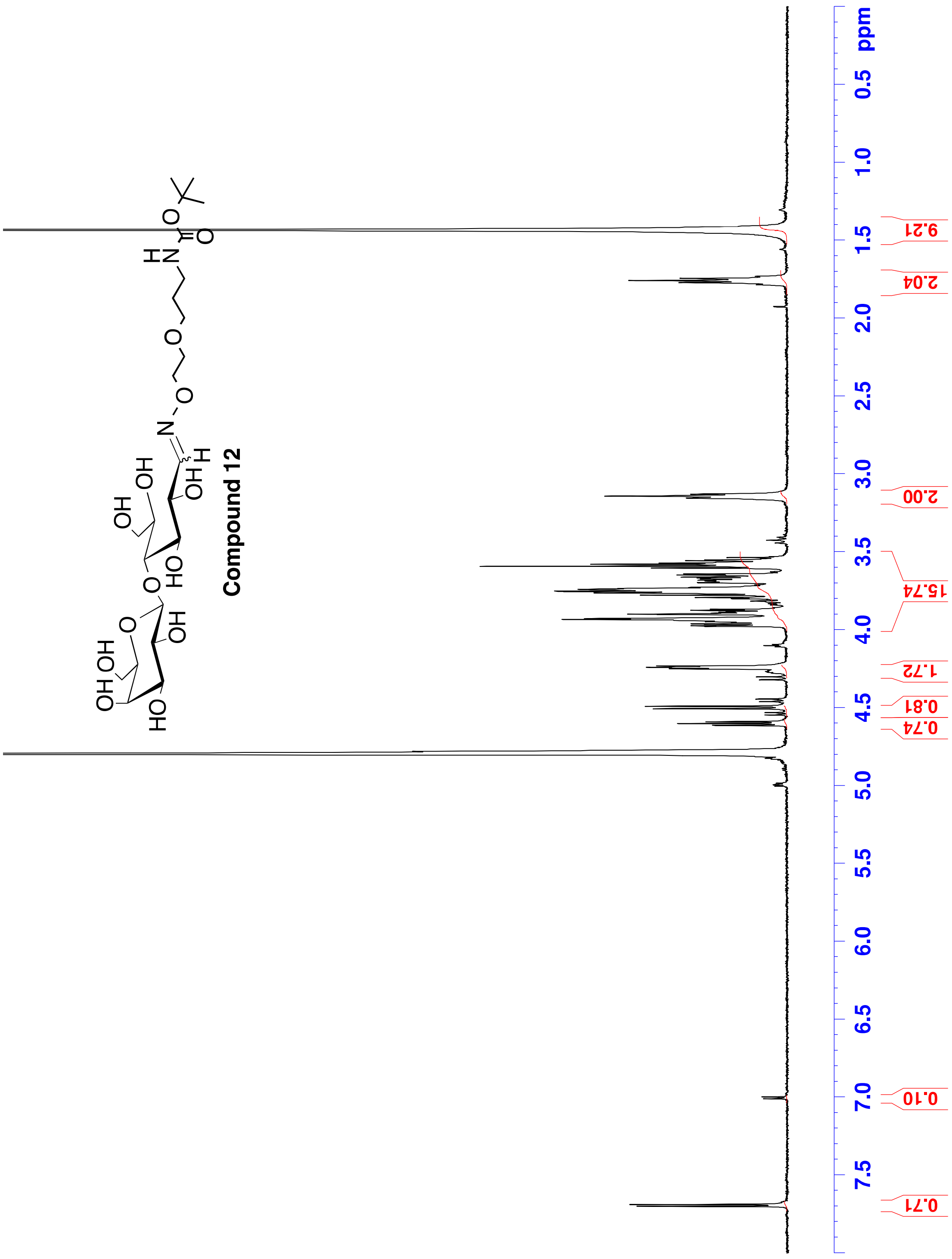

Figure S8: ^{13}C NMR of **Compound 12** (125 MHz, D_2O, internal MeOH standard).

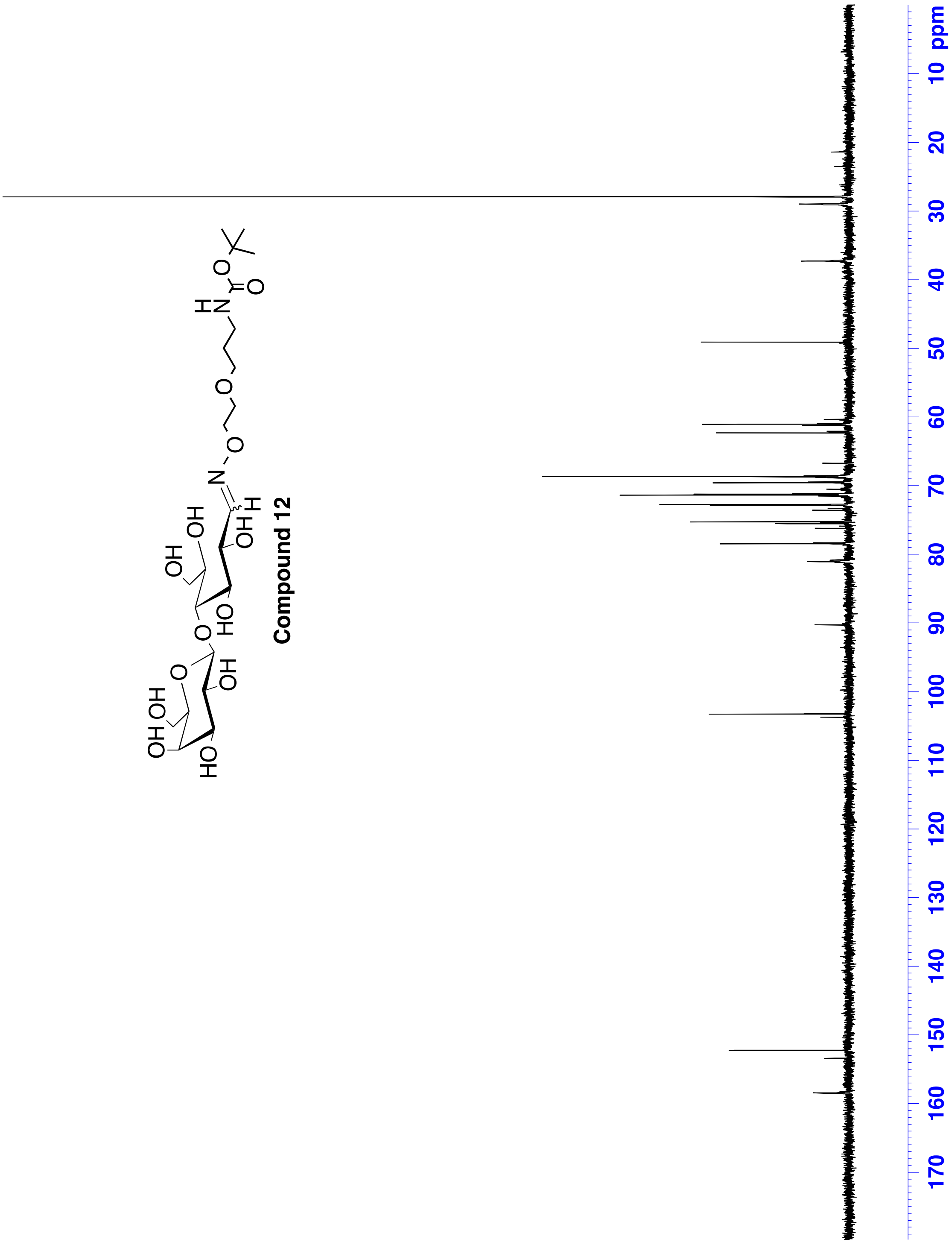

Figure S9: ^{1}H NMR of **Compound 13** (500 MHz, D_2O).

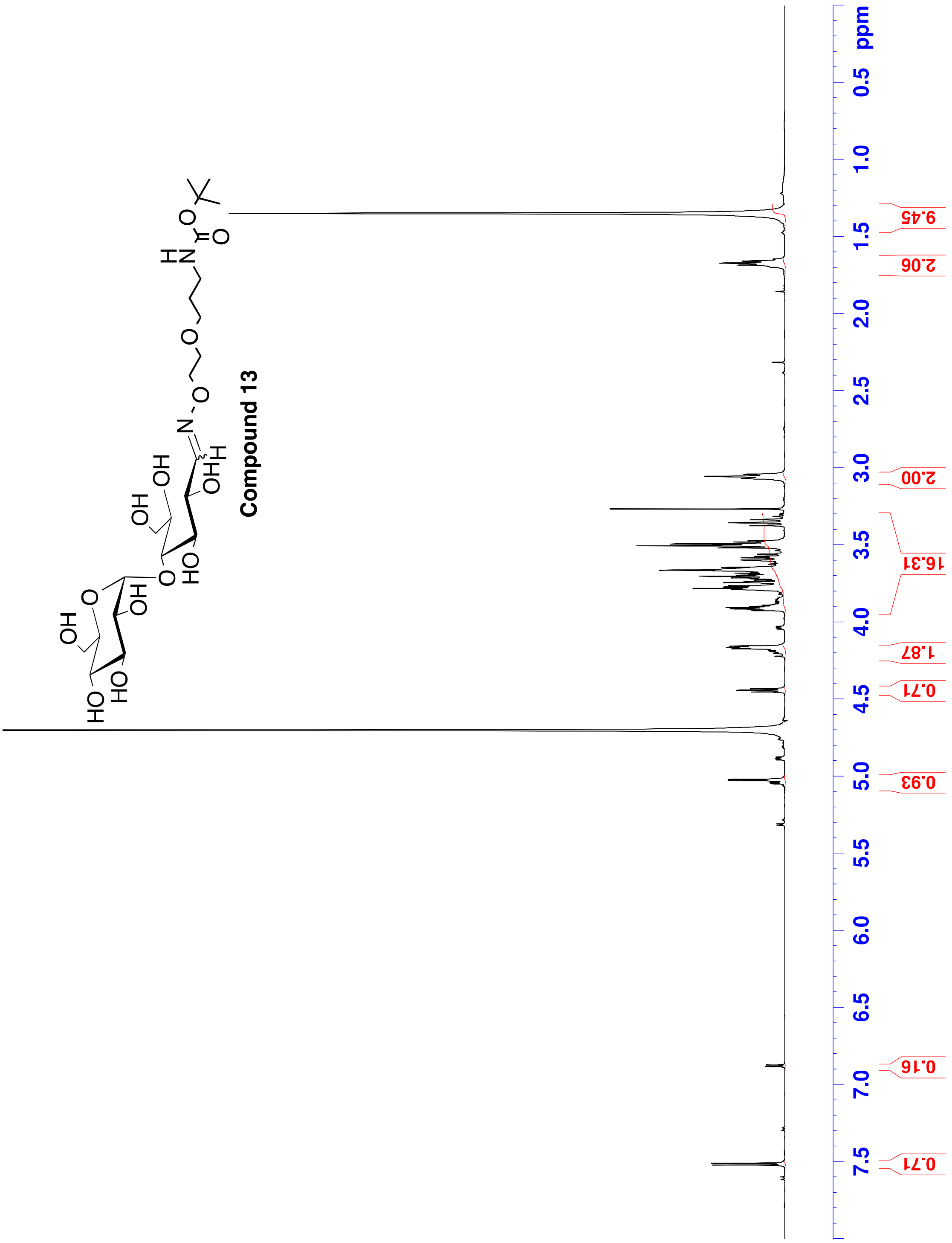

Figure S10: ^{13}C NMR of **Compound 13** (125 MHz, D_2O, internal MeOH standard).

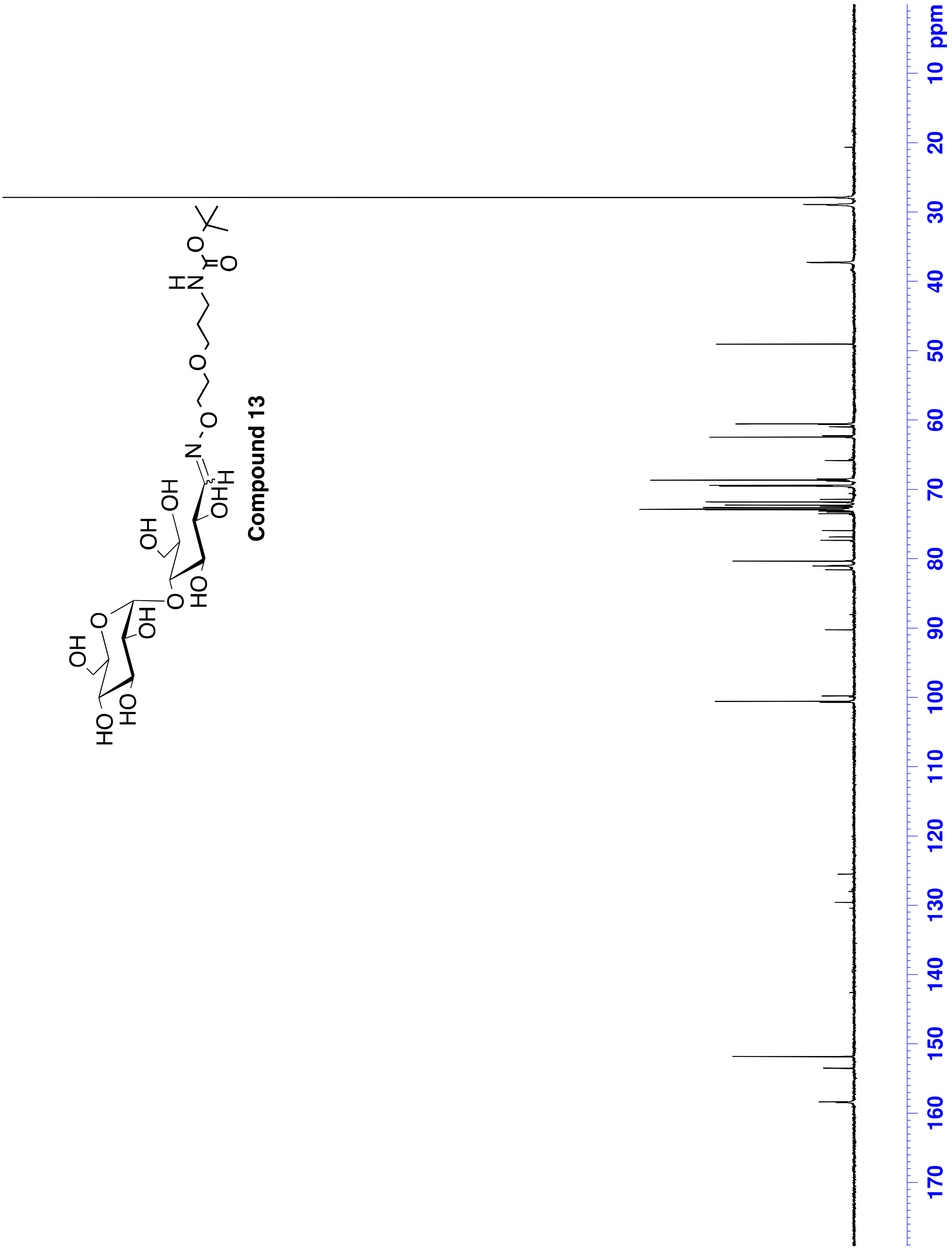

Figure S11: ^{1}H NMR of **Compound 14** (500 MHz, D_2O).

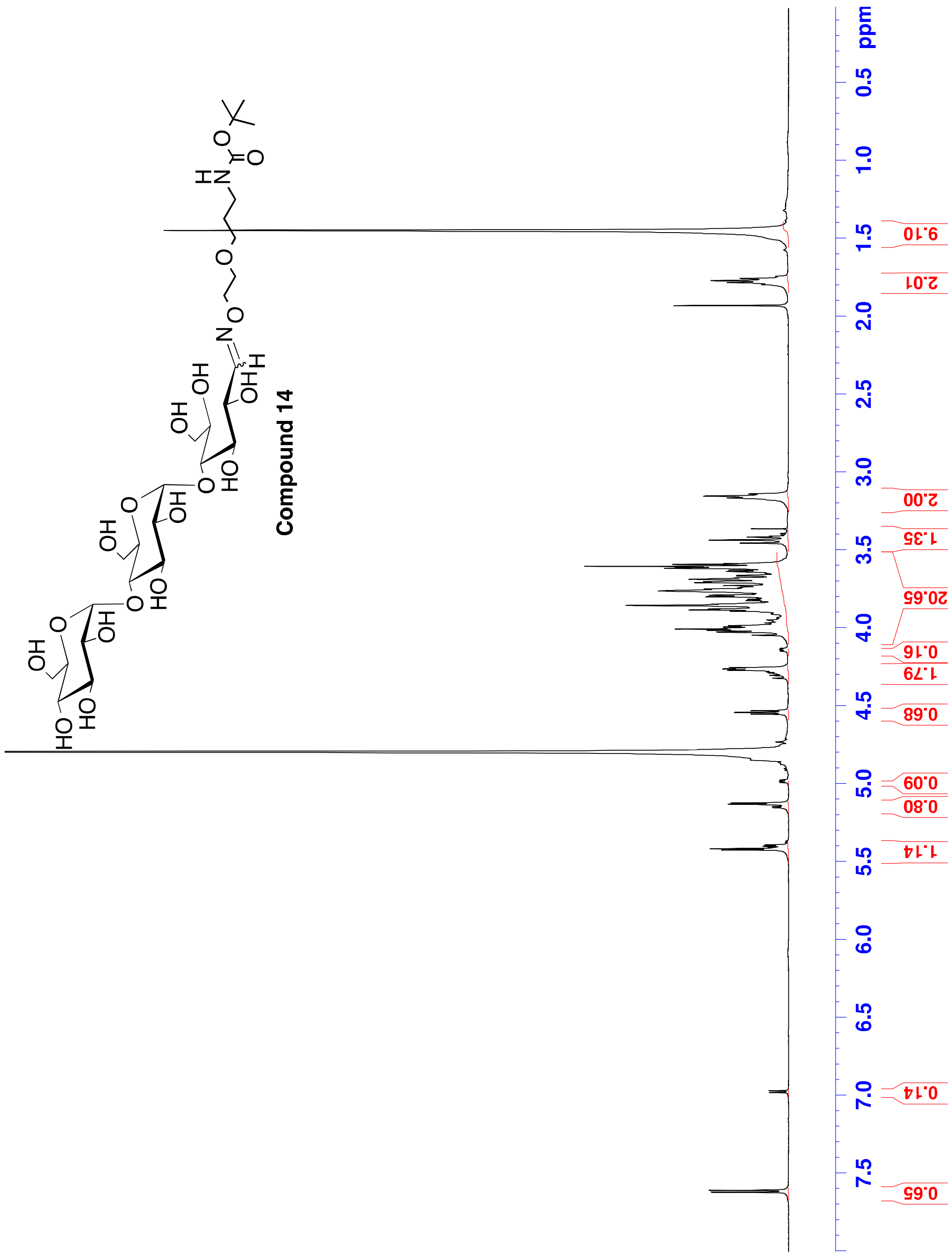

Figure S12: ^{13}C NMR of **Compound 14** (125 MHz, D_2O, internal MeOH standard).

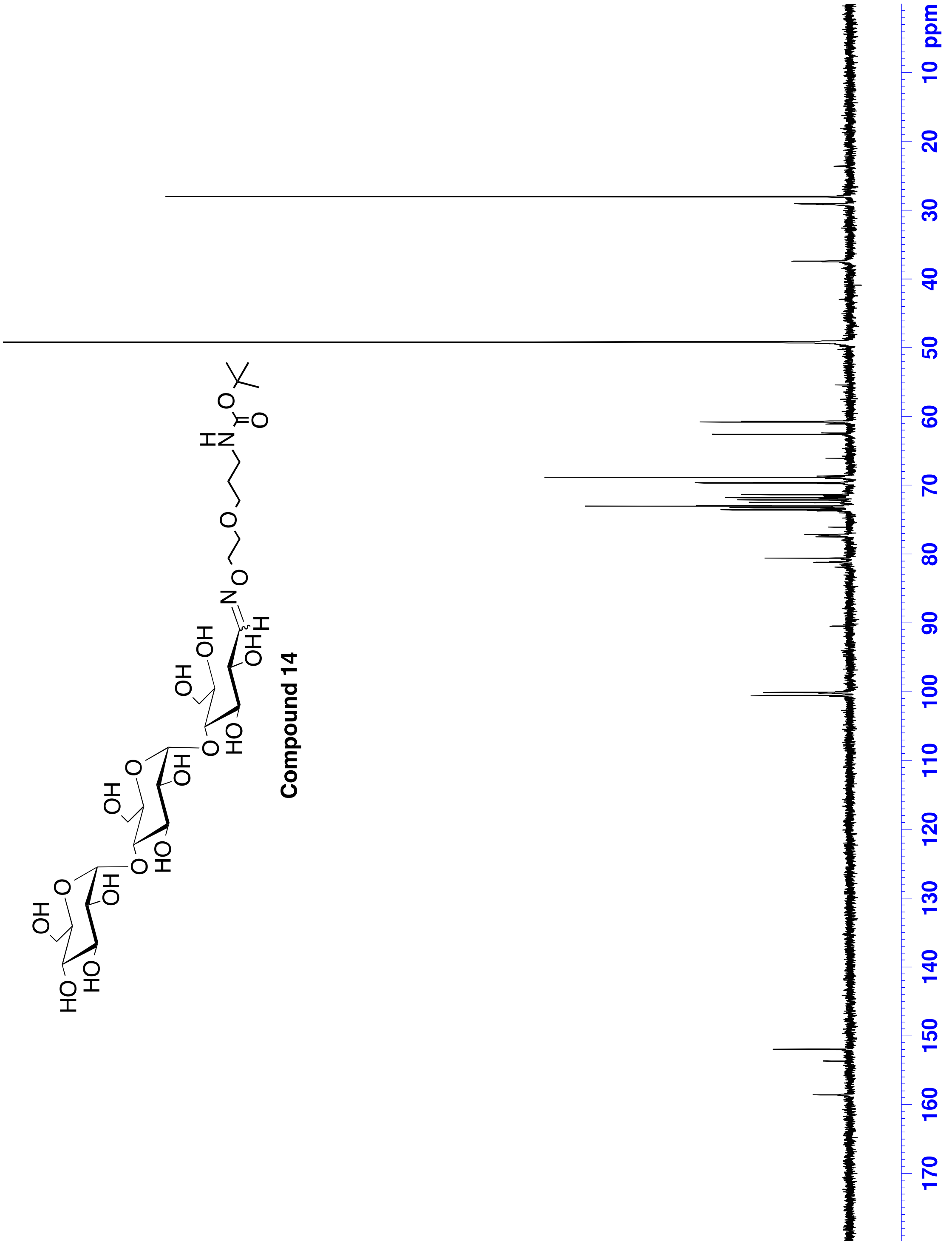

Figure S13: ^{1}H NMR of **Compound 15** (500 MHz, D_2O).

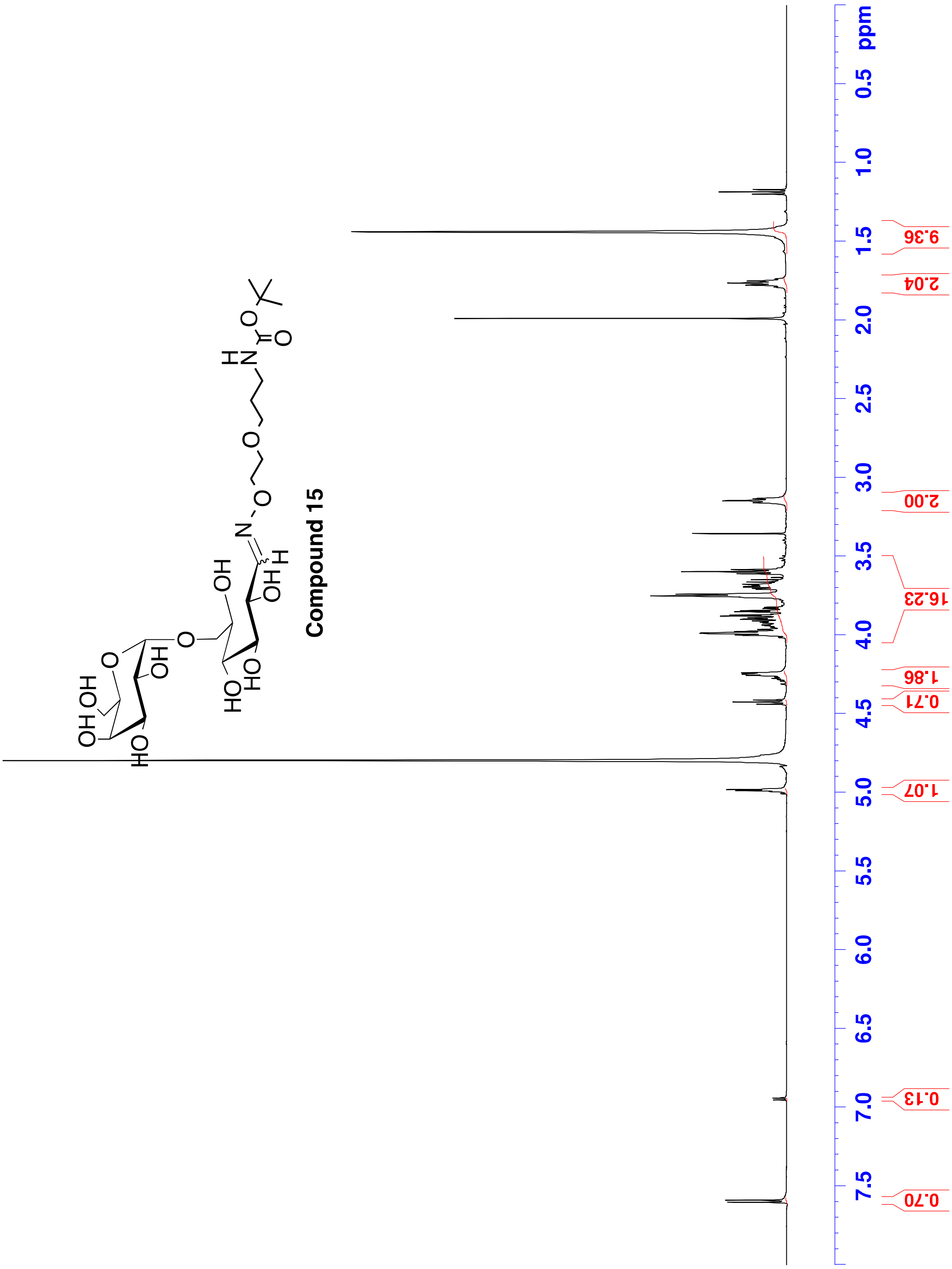

Figure S14: ^{13}C NMR of **Compound 15** (125 MHz, D_2O, internal MeOH standard).

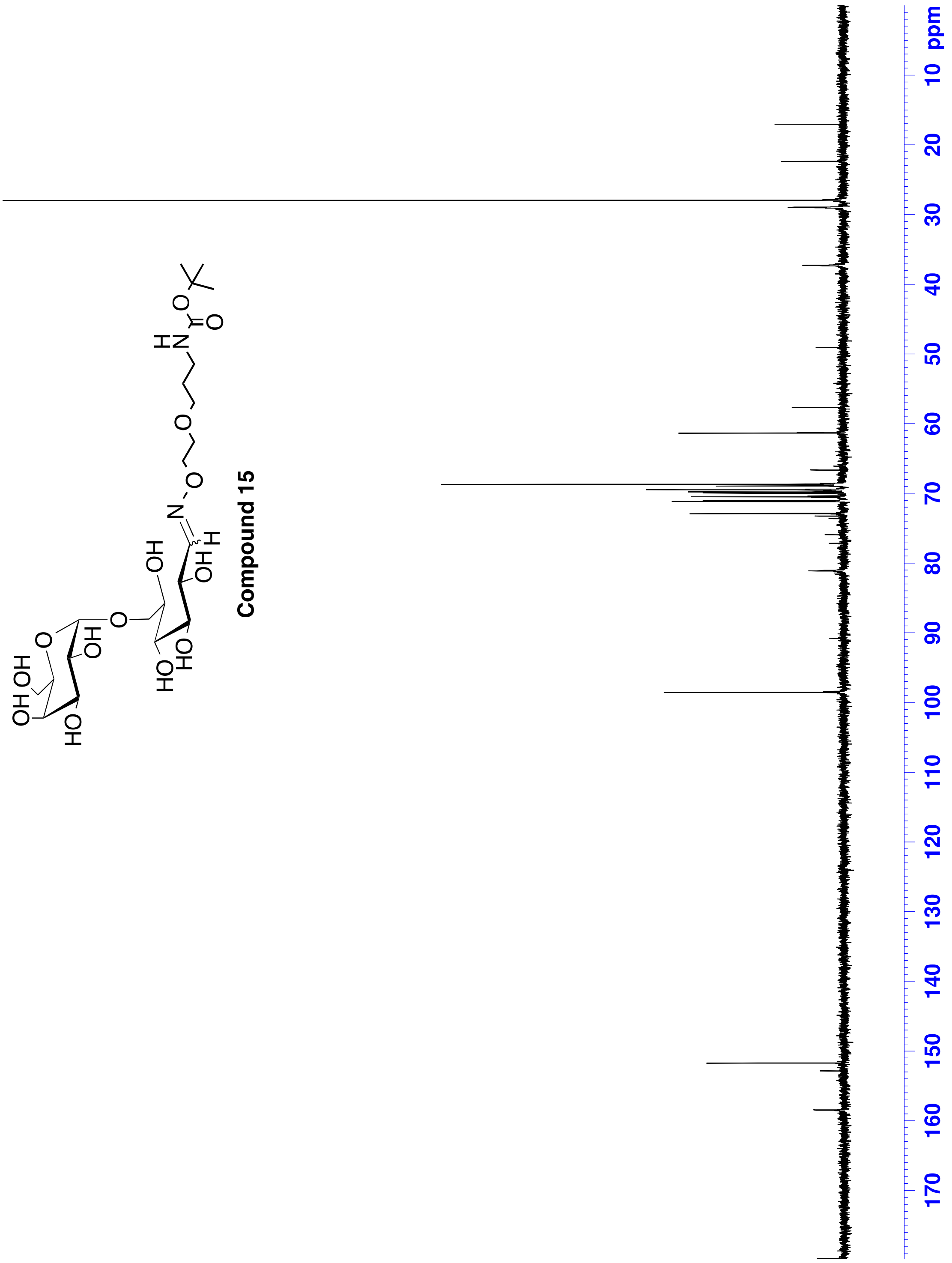

Figure S15: ^{1}H NMR of **Compound 17** (500 MHz, D_2O).

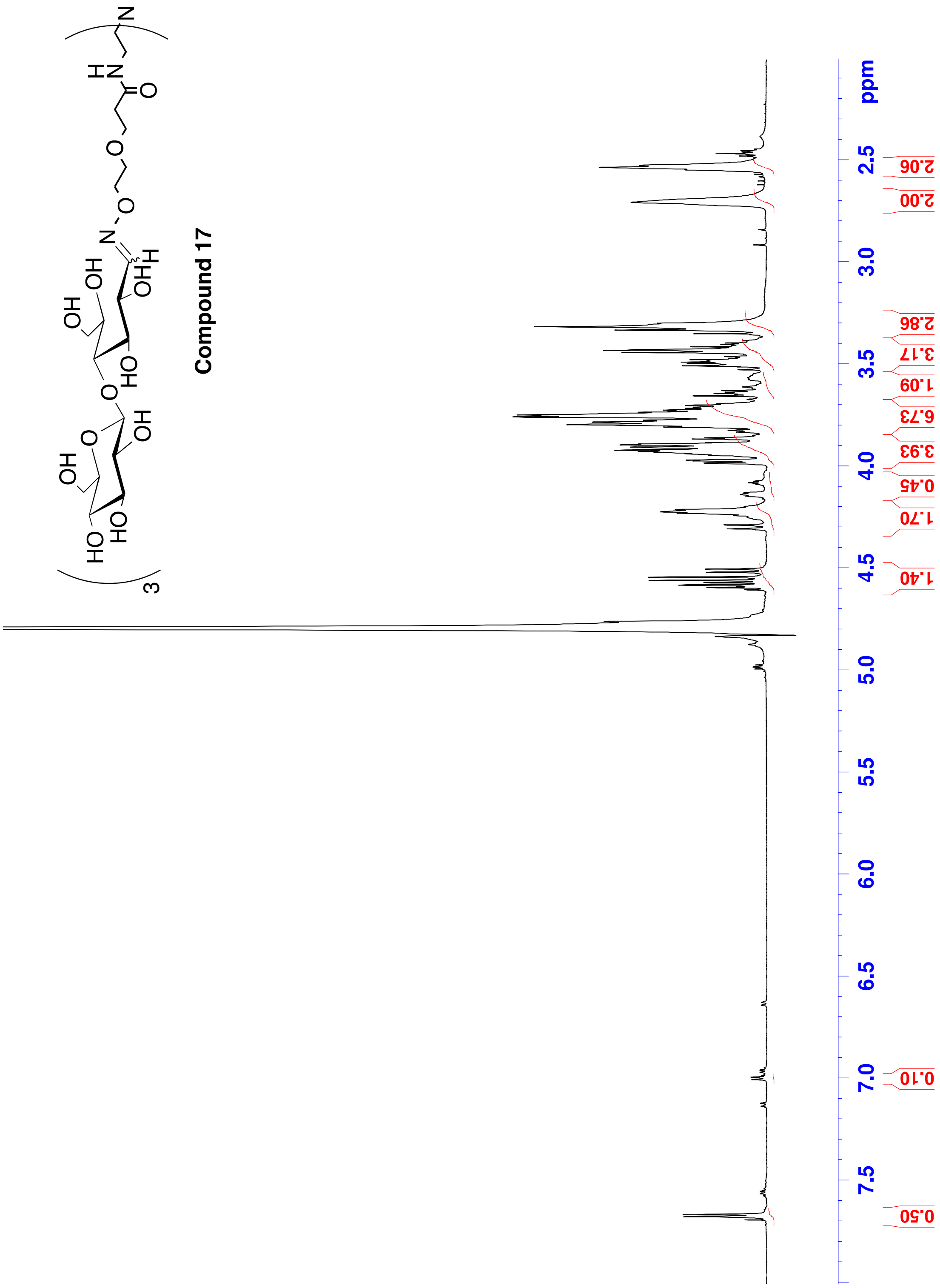

Figure S16: ^{13}C NMR of **Compound 17** (125 MHz, D_2O, internal MeOH standard).

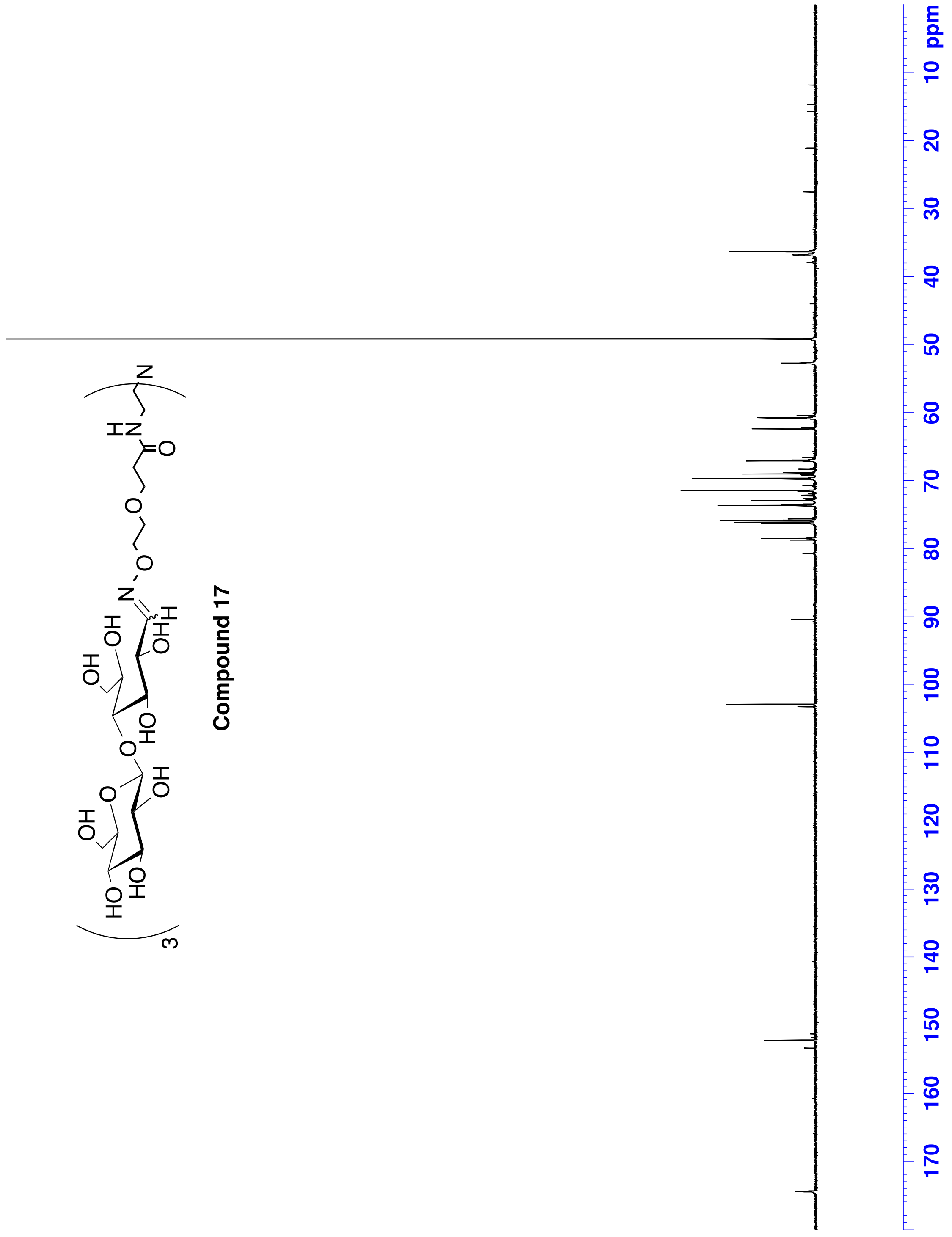

Figure S17: ^{1}H NMR of **Compound 20** (500 MHz, D_2O).

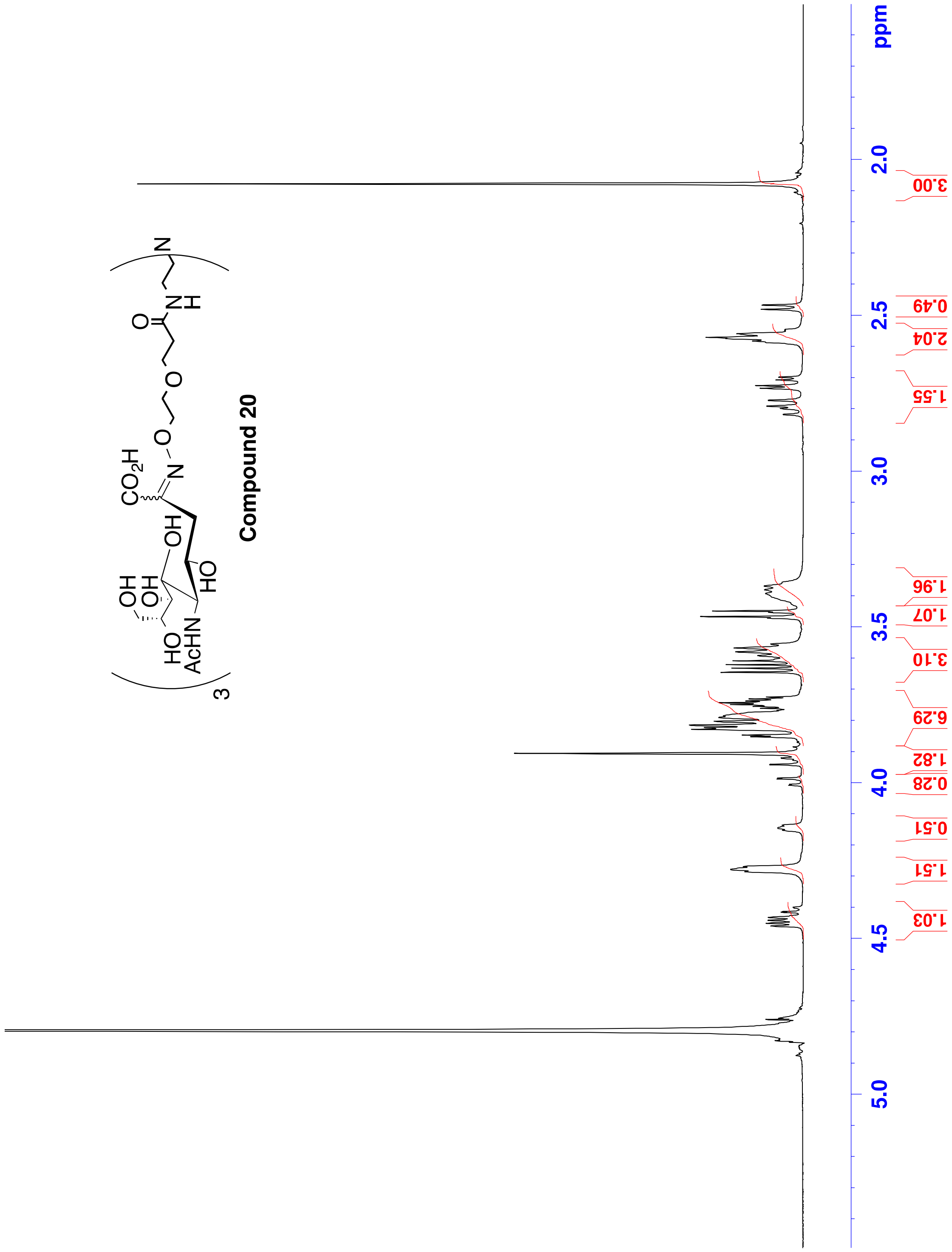

Figure S18: ^{13}C NMR of **Compound 20** (125 MHz, D_2O, internal MeOH standard).

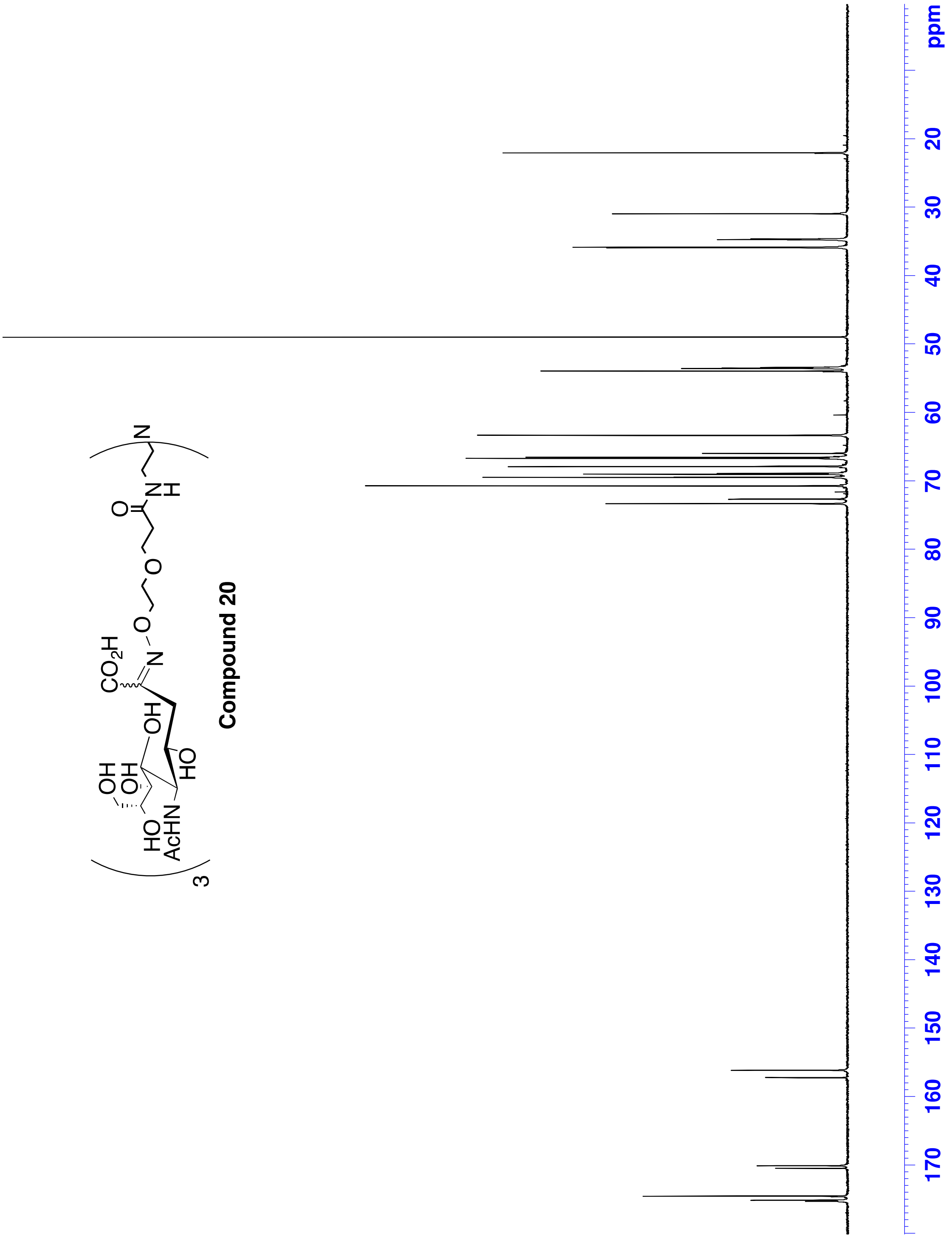

Figure S19: ^{1}H NMR of **Compound 21** (500 MHz, D_2O).

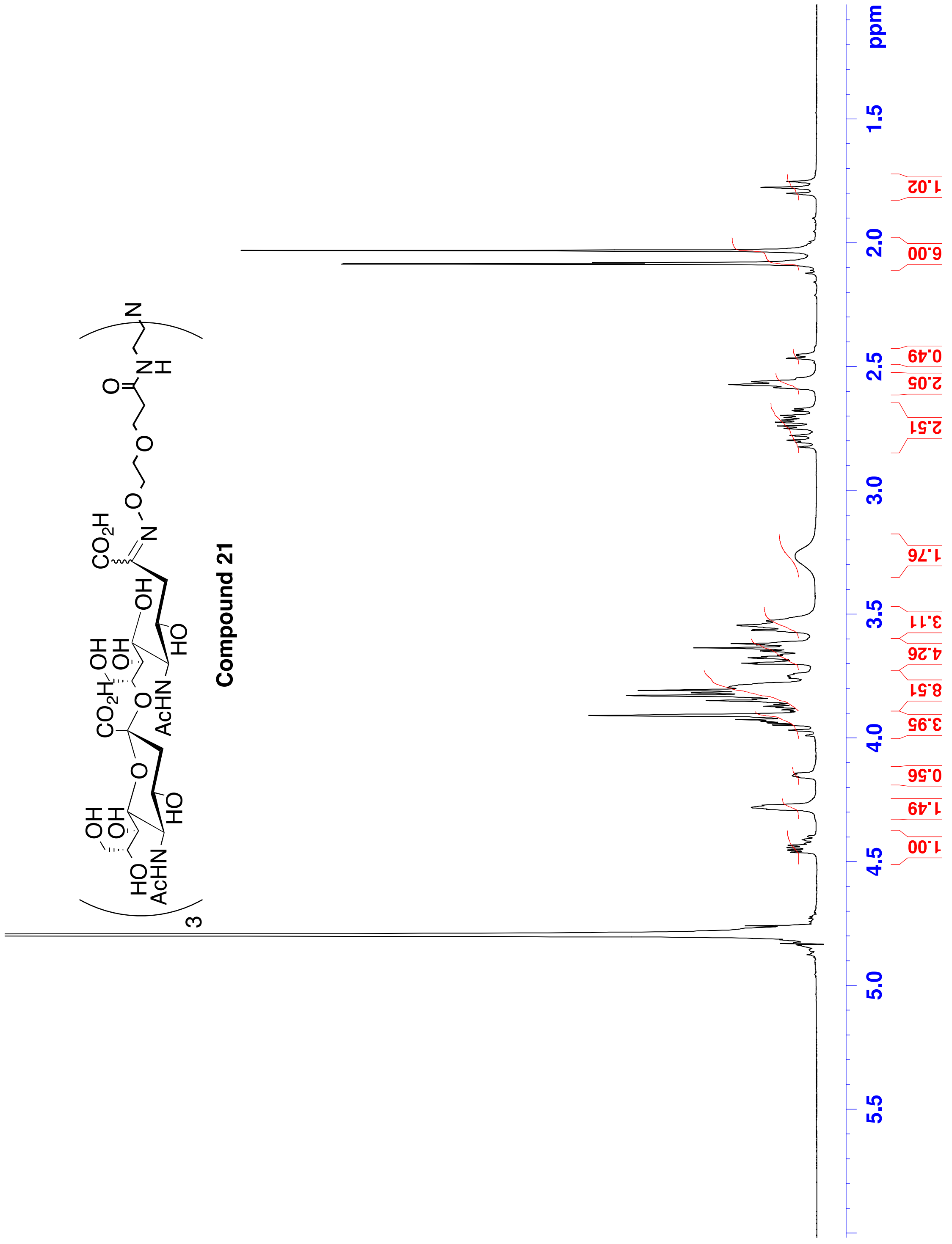

Figure S20: ^{13}C NMR of **Compound 21** (125 MHz, D_2O, internal MeOH standard).

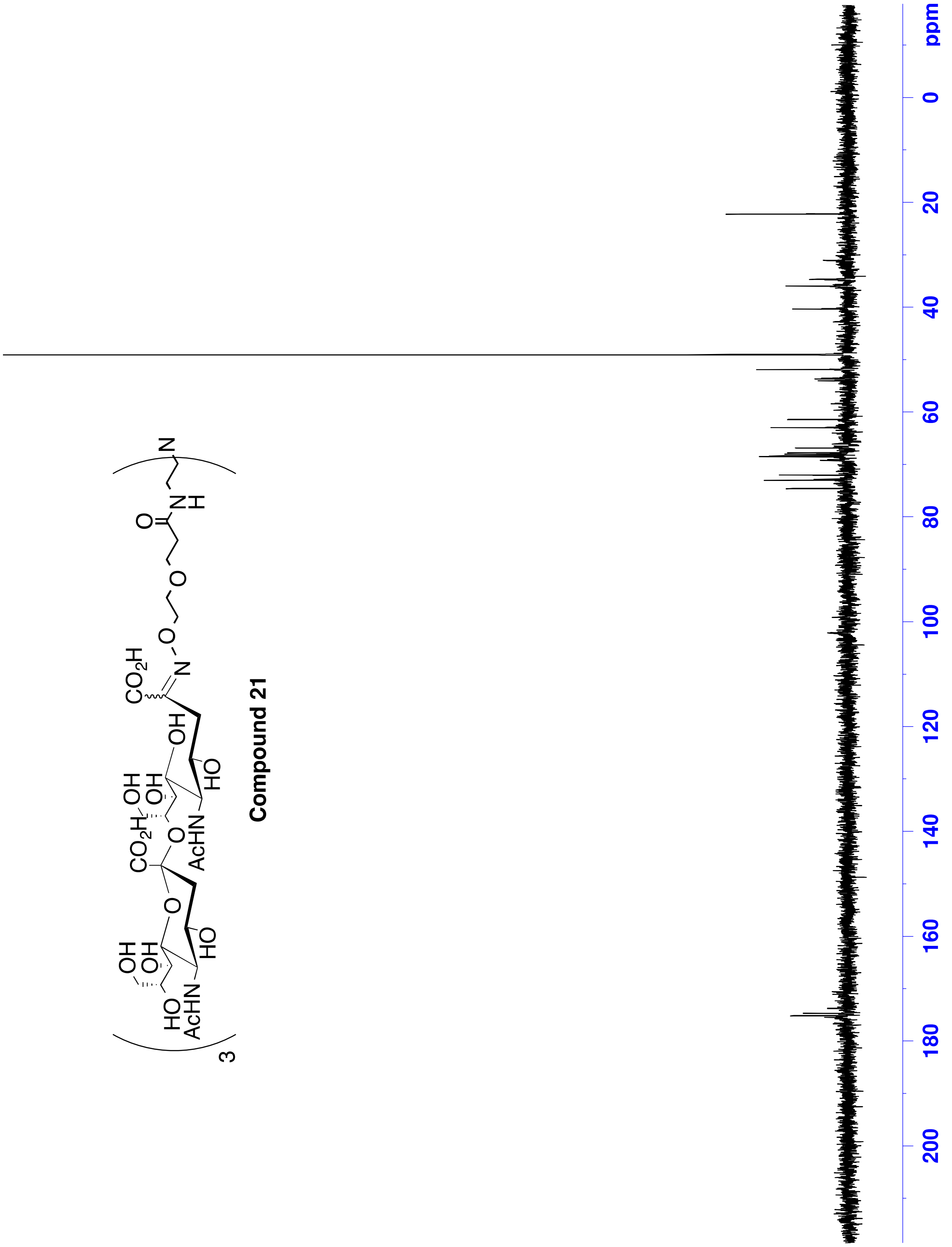

References:

1. Ray, G.J.; Siekman, J.; Scheinecker, R.; Zhang, Z.; Gerasimov, M.V.; Szabo, C.M.; Kosma, P. Reaction of oxidized polysialic acid and a diaminooxy linker: Characterization and process optimization using nuclear magnetic resonance spectroscopy. *Bioconjugate Chem.* **2016**, *27*, 2071-2080.
2. Thygesen, M.B.; Sauer, J.; Jensen, K.J. Chemoselective capture of glycans for analysis on gold nanoparticles: Carbohydrate oxime tautomers provide functional recognition by proteins. *Chem. Eur. J.* **2009**, *15*, 1649-1660.
